# Autoencoder/RandomForest–TabPFN for cross-cancer metabolomics: prostate and breast cancer diagnosis using paper spray and ion mobility-mass spectrometry techniques

**DOI:** 10.1093/gigascience/giag053

**Published:** 2026-05-07

**Authors:** Sven Hauns, Frederico G Pinto, Costerwell Khyriem, Ankita Singh, Azzat Al-Sadi, Talal Al Yazeedi, Rasheed Mohammad, Babacar Cisse, Timothy J Garrett, Mohammed Uddin, Nelson C Soares, Rolf Backofen, Omer S Alkhnbashi

**Affiliations:** Bioinformatics Group, Department of Computer Science, University of Freiburg, Georges-Köhler-Allee 101, 79110 Freiburg, Germany; Center for Applied and Translational Genomics (CATG), Mohammed Bin Rashid University of Medicine and Health Sciences (MBRU), Dubai Healthcare City, 505055 Dubai, United Arab Emirates; Institute of Exact Sciences, Federal University of Viçosa, Rio Paranaíba 38810-000, Brazil; Bioinformatics Group, Department of Computer Science, University of Freiburg, Georges-Köhler-Allee 101, 79110 Freiburg, Germany; College of Medicine, Mohammed Bin Rashid University of Medicine and Health Sciences (MBRU), Dubai Healthcare City, 505055 Dubai, United Arab Emirates; Center for Applied and Translational Genomics (CATG), Mohammed Bin Rashid University of Medicine and Health Sciences (MBRU), Dubai Healthcare City, 505055 Dubai, United Arab Emirates; Department of Computer Engineering, Hadhramout University, Hadhramout, P.O. Box 50512-50511, Yemen; Center for Applied and Translational Genomics (CATG), Mohammed Bin Rashid University of Medicine and Health Sciences (MBRU), Dubai Healthcare City, 505055 Dubai, United Arab Emirates; Department of Computer Science, College of Computing and Digital Technology, Birmingham City University, Birmingham B4 7XG, UK; Department of Neuroscience, Dubai Health, Dubai P.O. Box 505055, United Arab Emirates; Department of Pathology, Immunology, and Laboratory Medicine, University of Florida, Gainesville, FL Florida 32608, United States; Southeast Center for Integrated Metabolomics, Clinical and Translational Science Institute, University of Florida, Gainesville, FL 32610, USA; Center for Applied and Translational Genomics (CATG), Mohammed Bin Rashid University of Medicine and Health Sciences (MBRU), Dubai Healthcare City, 505055 Dubai, United Arab Emirates; College of Medicine, Mohammed Bin Rashid University of Medicine and Health Sciences (MBRU), Dubai Healthcare City, 505055 Dubai, United Arab Emirates; GenomeArc Inc, Mississauga, Ontario, Canada; Center for Applied and Translational Genomics (CATG), Mohammed Bin Rashid University of Medicine and Health Sciences (MBRU), Dubai Healthcare City, 505055 Dubai, United Arab Emirates; College of Medicine, Mohammed Bin Rashid University of Medicine and Health Sciences (MBRU), Dubai Healthcare City, 505055 Dubai, United Arab Emirates; Laboratory of Proteomics, Department of Human Genetics, National Institute of Health Doutor Ricardo Jorge (INSA), Lisbon, Portugal; Comprehensive Health Research Centre (CHRC), NOVA Medical School, University NOVA of Lisbon, Lisbon, Portugal; Bioinformatics Group, Department of Computer Science, University of Freiburg, Georges-Köhler-Allee 101, 79110 Freiburg, Germany; Signalling Research Centres BIOSS and CIBSS, University of Freiburg, Schänzlestr. 18, 79104 Freiburg, Germany; Center for Applied and Translational Genomics (CATG), Mohammed Bin Rashid University of Medicine and Health Sciences (MBRU), Dubai Healthcare City, 505055 Dubai, United Arab Emirates; College of Medicine, Mohammed Bin Rashid University of Medicine and Health Sciences (MBRU), Dubai Healthcare City, 505055 Dubai, United Arab Emirates

**Keywords:** metabolomics, prostate cancer, breast cancer, tabPFN, autoencoder, paper spray ionization mass spectrometry, artificial intelligence, cancer diagnostics

## Abstract

Accurate and rapid disease diagnosis, particularly in prostate cancer (PC) and breast cancer (BC), is critical for early intervention and improved patient outcomes. Metabolomic signatures represent a robust molecular framework for elucidating cancer-associated biochemical reprogramming. The use of artificial intelligence (AI) in biology in recent years has become widespread and promising. This study introduces a novel predictive method that integrates an Autoencoder, random forest-based feature selection and Tabular Prior-data Fitted Network (TabPFN) to achieve high diagnostic accuracy from metabolomics data of prostate and BC patients. The datasets were acquired using paper spray ionization mass spectrometry and flow injection-traveling-wave ion mobility-mass spectrometry of individuals diagnosed with PC and BC. When leveraging metabolomic profiling data from two distinct sources, PC urine and serum samples, the proposed model achieved an accuracy up to 98.75% in distinguishing diseased from healthy conditions. Additionally, we employed a BC dataset containing metabolic and lipidomic signatures acquired from core needle biopsies using a miniature MS platform coupled with PSI to assess the fidelity of our implementation across distinct cancer types. Our results on a well-characterized targeted dataset show that we can effectively reduce high-dimensional data into latent feature representations. At the same time, TabPFN captures tumor progression-related changes and feature interaction, thereby enhancing the possibility that the model will be a highly potent and effective tool for stage-specific diagnostic precision. Most existing machine learning approaches for disease diagnosis primarily rely on imaging, genomics, or clinical parameters, often overlooking the critical role of metabolites in identifying disease-specific biochemical signatures. By integrating metabolite-specific data with a robust deep-learning approach, this study demonstrates the transformative potential of AI in metabolomics-based diagnostics. The proposed model offers scalability and versatility, with applications extending beyond oncology to a much broader disease profiling aspect. These findings emphasize the value of combining multi-source metabolomic data with deep learning to advance personalized medicine and enhance diagnostic efficiency in clinical practice.

## Introduction

The accurate and timely diagnosis of diseases, particularly cancers, is crucial for enhancing patient outcomes and facilitating early intervention. Although traditional diagnostic approaches, such as histopathological examinations, imaging modalities, and biochemical assays, remain the cornerstone of definitive disease diagnosis, they are often invasive, time-consuming, and costly. Moreover, these methods may lack the sensitivity and specificity necessary for early detection or for distinguishing between disease subtypes. This underscores the need for innovative, less-invasive diagnostic approaches that combine advanced molecular profiling techniques with robust computational frameworks to transform disease detection and management [1, 2].

Metabolomics, i.e. the comprehensive capture and analysis of small molecules in biological systems, has emerged as a powerful tool in modern diagnostics. Leveraging high throughput mass spectrometry (MS) platforms, such as paper spray ionization mass spectrometry (PSI-MS) and flow injection-traveling-wave ion mobility-mass spectrometry (FI-TWIM-MS), metabolomics enables the identification of key biomarkers associated with disease states, offering insights into underlying molecular mechanisms.

Conventional metabolomic workflows, particularly those based on liquid chromatography coupled with mass spectrometry (LC-MS), often necessitate comprehensive sample preparation and protracted chromatographic separation. These constraints limit their applicability in clinical settings, where expeditiousness and simplicity are paramount. In contrast, the emerging techniques of FI-TWIM-MS and PSI-MS represent rapid and cost-efficient high-throughput alternatives that eliminate the need for complex and time-consuming chromatographic separation while maintaining analytical sensitivity.

PSI-MS-based metabolomics has gained traction for rapidly analyzing biofluids such as urine, blood, and serum without complex sample preparation [3–5]. For example, PSI-MS has been used to identify distinct metabolic signatures in prostate and breast cancers (BCs), achieving good diagnosis by integrating advanced chemometric and traditional analyses [6–9]. Additionally, this technique demonstrates considerable potential for cost-efficient, labor-efficient, and rapid disease diagnosis. Flow injection-traveling wave ion mobility-mass spectrometry (FI-TWIM-MS) is an analytical technique that combines the rapid, chromatography-free sample introduction of flow injection (FI) with the gas-phase ion separation capabilities of traveling wave ion mobility (TWIM) and high-resolution mass analysis of mass spectrometry. FI-TWIM-MS was applied to discriminate between healthy and prostate cancer (PC) patients using serum samples [10].

Despite these technological advancements, metabolomic data remains high-dimensional and subject to noise, necessitating sophisticated analytical approaches to extract salient patterns [11, 12]. Artificial intelligence (AI) and deep learning techniques present robust solutions to this challenge. Autoencoders (AEs) compress input features into latent representations, effectively capturing complex interrelations within the data. Furthermore, transformer-based models such as TabPFN [13], which are pre-trained on extensive tabular datasets, facilitate robust classification even when sample sizes are limited [9, 10]. Previous research has already shown promising results pre-selecting a limited number of features and applying logistic regression [14].

The integration of AI with rapid MS technologies fosters the development of scalable, real-time, and noninvasive diagnostic frameworks. However, challenges persist concerning the standardization of protocols, the management of heterogeneous data sources, and the achievement of clinical scalability [11–15].

To address these challenges, we propose an innovative approach that combines AE-based compression, feature selection based on random forests (RFs), and, as a final step, the TabPFN classifier to analyze metabolomic data from two PC studies; one employing PSI-MS (urine) [6, 7] and the other utilizing FI-TWIM-MS (serum) [10]. Additionally, we included one BC metabolic profile based on PSI-MS data to examine the utility of TabPFN on a disease similar to PC but completely unrelated [16, 17]. This multi-source, AI-integrated approach has achieved classification accuracy of up to 98.75%, underscoring its potential to advance personalized medicine and enhance clinical decision-making in the field of oncology.

## Method

### Datasets

Patients and 42 healthy controls (total of 103 samples) [10] and 237 metabolite features per sample. The serum samples underwent processing through a cold solvent extraction protocol, followed by phase separation to eliminate lipids and proteins. FI-TWIM-MS facilitated rapid analysis, with each sample being processed in ∼6 min, including wash runs, while concurrently separating ions based on both mass-to-charge ratio (m/z) and collision cross section. The use of ion mobility enhanced resolution within complex mixtures contributed high-quality input for downstream classification tasks. The second dataset consists of urine-based metabolic profiles, which were acquired using PSI-MS. This data was sourced from previously published studies [6, 7] that involved urine samples obtained from 40 PC patients, as well as from 40 healthy control subjects. The PSI-MS technique facilitated rapid and direct ionization of dried urine spots with minimal sample preparation, yielding comprehensive mass spectra across the m/z range. After filtering, the dataset contained 784 metabolite features per sample. These features represent intensity values at different m/z. In contrast, the third dataset utilized PSI-MS to analyze core-needle breast biopsies [17]. It included 692 samples, with 204 malignant and 488 benign cases, each characterized by 494 metabolite features, obtained using a miniature MS platform coupled with PSI, the MiniMaP platform. The spectra, covering an m/z range of 500–1000, mainly reflected lipid profiles annotated by histopathological examination. This dataset was used to evaluate the cross-disease and cross-platform generalizability of the AE/RF–TabPFN approach. All datasets were preprocessed using L₂-normalization to standardize feature scales before model training.

### General strategy for model selection

To evaluate the quality of our predictions, we utilize nested 10-fold cross-validation (CV) to ensure a fair and robust performance evaluation for our hyperparameter optimization. The final models are then created by averaging the hyperparameter result of the nested CV (latent dimension or number of selected features) and then retraining the model on the data used for the inner CV, while evaluating it on the outer CV to guarantee an independent test set.

**Figure 1:**
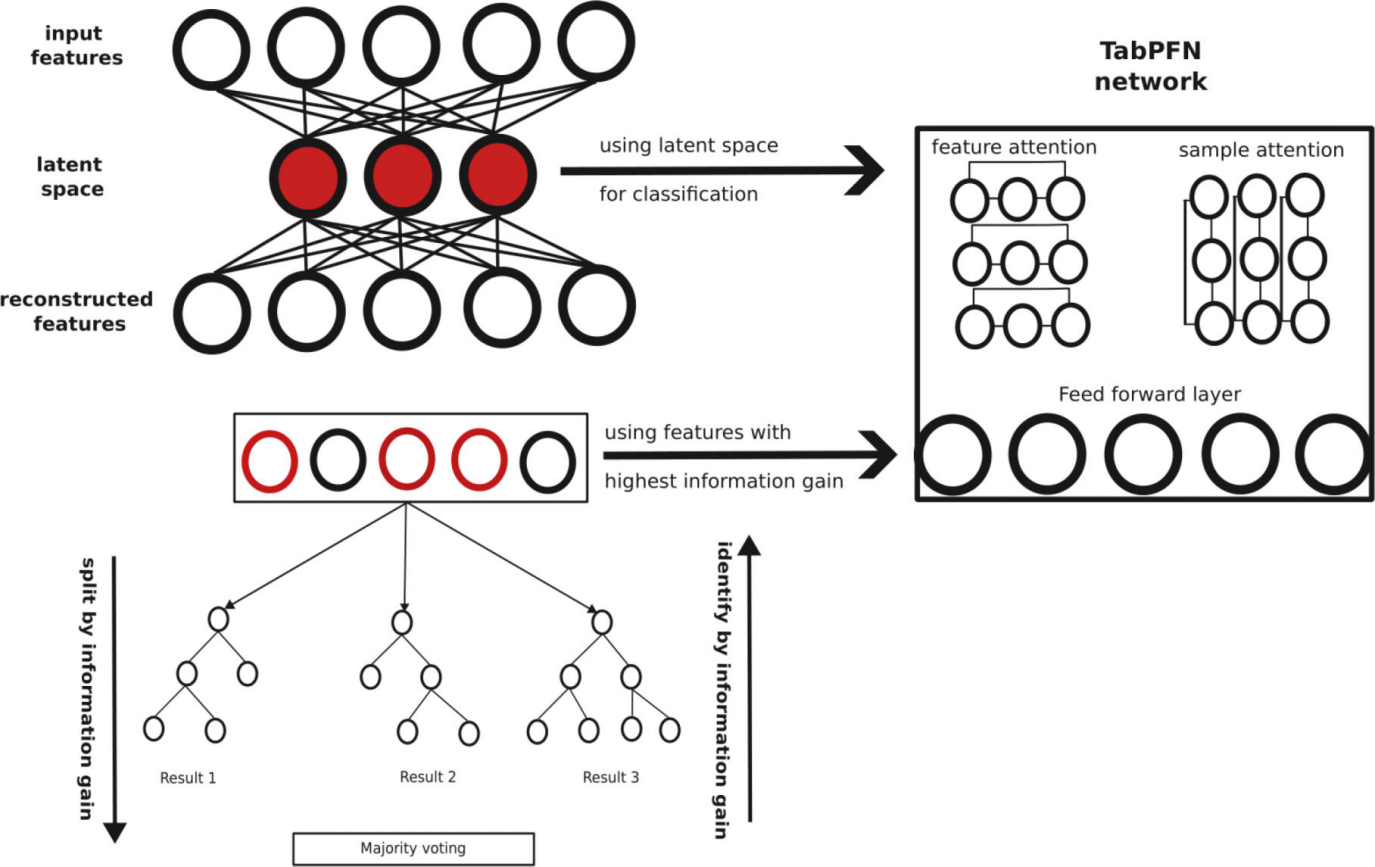
It shows the principal feature selection workflow employed to prevent overfitting. An AE is used to create a latent dimension for classification. This helps with reducing redundant information and de-noises the data for further processing. Alternatively, we use the information gained based on RFs to assess which feature to select for further processing.

Since metabolomic datasets often exhibit high dimensionality but contain relatively few samples, we explore two techniques to select informative input features and mitigate the risk of overfitting. These techniques also help focus on the most salient input features, while disregarding redundant information in the temporally structured metabolomic data, and improve the classification performance. Figure 1 provides an overview of this feature selection pipeline, showing the two main strategies: AE-based latent feature extraction and RF-based feature importance estimation. Those two strategies were used prior to final classification with the TabPFN model.

Our model was applied as the first step of the feature reduction approach to provide TabPFN with a smaller set of important features. For this first step, we use two different methods. The first is a simple feature selection process based on Shannon entropy and information gain, calculated from RF splits. Here, Shannon entropy is used at each node of a tree to determine how well a potential feature splits the data into distinct groups, measuring the uncertainty within each subset of samples. A split that maximizes information gain and hence minimizes entropy is chosen. For trees created in this manner, feature importance can be calculated by summing the total information gained by that feature across all data splits and all trees. This approach enables identifying a variable number of highly informative input features and assess how the final prediction accuracy changes with different numbers of selected features.

The second method employs an AE, where the latent space serves as the feature extraction layer for subsequent classification tasks. A latent embedding was used as a compressed representation of the input features and later to analyze the importance of specific input features. To ensure the AE effectively captures relevant information, we condition it on both input reconstruction and final prediction by combining these objectives into a single loss function. Additionally, Shapley values were employed to identify which input features have the greatest impact on constructing the AE’s latent space.

The final classification is then undertaken to train the TabPFN [13] model on the preselected optimal features. Due to being pre-trained on input data described by up to 500 features, our feature reduction methods effectively transform the original input space to a dimension TabPFN can make best use of. Furthermore, it allows assessing feature importance and thus explainability, which is critical for acceptance of the method by medical professionals.

### Architecture and training

Since the data is very sparse, we rely on a pre-trained model to enable reliable classification while avoiding overfitting. We combine feature selection, based on RF feature importance, and feature compression using AEs with TabPFN for optimal classification results. The performance is evaluated by first fitting TabPFN to the data on the training fold and predicting the output on the test folds. In addition, to gain more insight into the structure of the feature space, we also applied a RF-based approach for direct feature selection for classification and to gain insight into the importance of the input features. To overcome the possibility of overfitting, sampling was applied to ensure an even distribution of positive and negative samples in every fold.

To ensure a fair evaluation of our method, all hyperparameter optimizations were run on a nested 10-fold CV. The returned hyperparameter were averaged and the final model retrained and evaluated on the test sets of the outer CV split. All baseline methods were trained for 100 epochs using an Adam optimizer and evaluated using 10-fold nested CV when we make use of hyperparameter optimization.

### Comparison to baseline

We employ multiple baseline methods to compare the combination of TabPFN with compression and feature selection against a selection of deep learning methods trained using the same compression pipeline. The first baseline model is a 1D CNN that expands the input channel dimension to 128 through three convolutional layers with kernel sizes of 2, followed by average pooling. This design allows us to effectively utilize small feature-selection sets and compressed representations. The second baseline model is a basic multilayer perceptron (MLP) consisting of three layers with ReLU activation functions and batch normalization. The third deep learning baseline model, which we refer to as MLP-surv due to its resemblance to the general architecture of DeepSurv [18], comprises three layers with alternating linear layers, batch normalization, and dropout.

### Input feature importance

**Figure 2:**
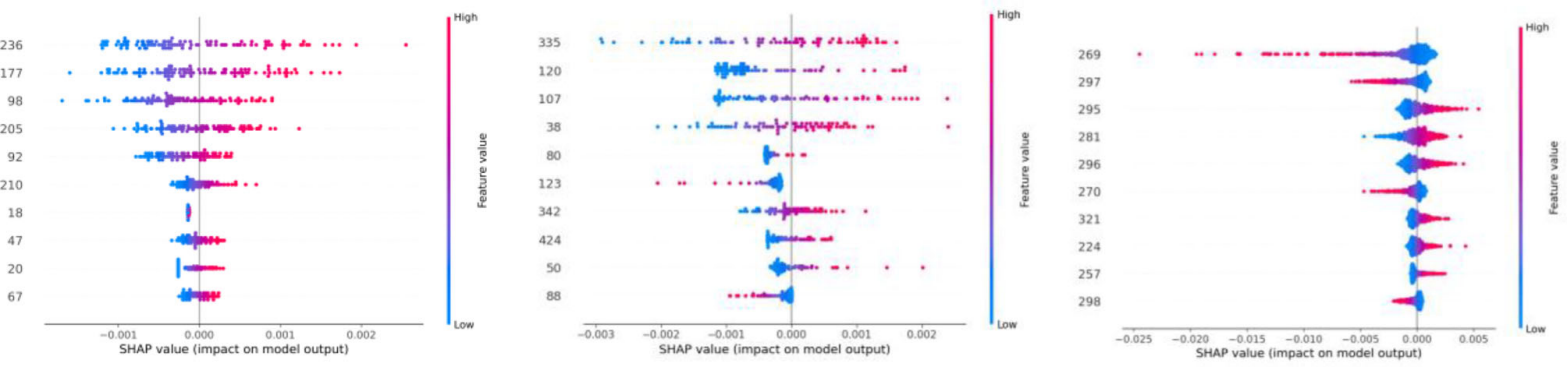
It shows the most important input features determined by SHAP values on the two datasets. The most important features for both datasets are spread throughout the dataset, illustrating the importance of carefully selecting the input features to be used for classification later on. Low importance is shown in blue shades, while high importance in red shades.

To enhance model interpretability and improve feature selection, two complementary approaches were applied: RF-based feature selection and AE-driven latent space analysis (as seen in Fig. 2). First, RFs were utilized to rank input features according to their importance in classification. Feature importance was determined using Shannon entropy and information gain, calculated from RF splits. This enables assessing the effects of different feature subsets on classification accuracy by selecting several and the most salient features. An AE-based latent space was incorporated to extract meaningful representations from high-dimensional metabolomic data. The AE was trained to reconstruct input data, while concurrently optimizing for classification performance. To interpret which input features contributed most significantly to the latent space, SHAP values were applied, which quantify the impact of each feature on the encoded representation. By combining these two methods, the aim was to identify the most relevant biomarkers, while minimizing noise and redundancy in the dataset. This feature selection strategy was implemented before training the TabPFN classifier to ensure that the most informative variables only contributed to the model predictions. Unlike RF classifiers, AEs allow us to create a latent space from the entire input sequence. The model automatically learns a representation that captures all vital information for the final classification.

## Results and discussion

### Our deep-learning model achieves high accuracy on a diverse set of tasks

For both datasets, we test different feature pre-processing methods to assess the best way of dealing with high-dimensional data with a low number of samples. The first is AE-based, where the input feature vectors are mapped to a latent space with a lower dimension, and the features of the latent space are used as input for TabPFN. The second is based on direct feature selection, which is performed using the estimation of feature importance by a RF classifier (see Methods). We thus term the different models AE-TabPFN-FS or RF-TabPFN-FS, where the first part determines the used method for feature selection (AE = autoencoder, RF = random forest), and FS describes the final TabPFN input feature size. For the FI-TWIM-MS dataset RF-TabPFN-210 results in an accuracy of 91% (±9.4), with a weighted F1-score of 0.91 (±0.0965) and a receiver operating characteristic area under the curve (ROC-AUC) of 0.95 (±0.06468). For the PSI-MS dataset, pre-selecting features using a RF and then fine tuning TabPFN (RF-TabPFN-236) creates an accuracy of 98.75% (±3.75), a weighted F1-score of 0.99 (±0.038) and a ROC-AUC of 1.0 (±0.0). Though only a smaller number of features were removed, it still outperforms TabPFN on the complete feature set, so combining TabPFN with feature selection is superior in our type of data (see Table 1). For the BC dataset [17], using the most important 170 features RF-TabPFN-170 reaches an accuracy of 90% (±4.098) with an ROC-AUC of 0.94 (±0.0381) and F1-score of 0.90 (±0.04311). Similar results are achieved with AE-TabPFN-240 with an accuracy of 89% (±1.9) and an ROC-AUC of 0.92 (±0.0339). Here, the AE provides more stable results over all folds.

**Table 1:** We see a general improvement in classification performance throughout all metrics when applying feature selection or transformation before using TabPFN.

experiment	TabPFN	RF/AE-TabPFN
FI-TWIM-MS-dataset	Accuracy: 0.88 ROC-AUC: 0.93 F1: 0.88	Accuracy: 0.91 ROC-AUC: 0.95 F1:0.91
PSI-MS-dataset	Accuracy: 0.975 ROC-AUC: 1.0 F1: 0.97	Accuracy: 0.99 ROC-AUC: 1.0 F1:0.99
Breast cancer dataset	Accuracy: 0.91 ROC-AUC 0.93 F1-score of 0.90	Accuracy: 0.90 ROC-AUC 0.94 F1-score of 0.90
Combined datasets	Accuracy: NA^a^ ROC-AUC: NA F1: NA	Accuracy: 0.94 ROC-AUC: 0.99 F1: 0.94

a The combination of both datasets needs preliminary feature selection, due to different feature dimensions. Therefore, TabPFN cannot be executed solely on the combined datasets.

Finally, we compare our method with standard machine learning methods on the same data split and find an improvement in our method over the alternatives. For the complete evaluation, see Fig. 4.

### Insights from the determined feature importance

We utilized SHAP values for two datasets to identify the most critical input features. Figure 2 highlights the distribution of feature importance across these datasets. In the FI-TWIM-MS dataset, which comprises PC urine samples, The most predictive features were predominantly located within the middle m/z range. This particular region is likely to include lipid-based metabolites such as phosphatidylcholines and triacylglycerols. These two molecules have been previously reported to be indicative of cancer metabolic reprogramming [19–21], pro-tumorigenic signaling [20, 21], resistance [23, 24], and metastasis [25, 26]. Particularly in PC, elevated phosphatidylcholines in exosomes from hormone-sensitive PCa cell lines LNCaP correlate with aggressive disease. Conversely, lysophosphatidylcholines in blood are linked to better prognosis. Since the feature analysis is based on m/z ranges rather than annotated metabolites, mechanistic insights cannot be directly derived from this step. The concentrated nature of these critical features indicates the presence of a relatively compact biomarker signature in urine, thereby facilitating streamlined diagnostic applications. The PSI-MS dataset exhibits a more concentrated set of features, and thus the model RF-TabPFN-236 shows the best result of 98.75% classification accuracy. Though only a smaller number of features were removed, it still outperforms TabPFN on the unfiltered feature set. So, combining TabPFN with feature selection is superior in our type of data. This pattern signifies a more complex and heterogeneous metabolic profile, likely attributable to the varied biological processes represented in serum. Consequently, the classification model must extract relevant signals from a wider range of metabolites to ensure the maintenance of high diagnostic accuracy.

The SHAP-based feature importance analysis provides interpretable insights into the model’s decision-making process, facilitating the identification of biomarkers with high clinical relevance. These results highlight the flexibility of the AE-TabPFN model, which can adapt to the unique feature distributions of each dataset. This adaptability is crucial for enabling precise diagnostics across diverse metabolomic datasets. The improvement gained by using feature selection and compression is also evident in all classification metrics, as shown in Table 1.

### Exploring the relationship between selected features and accuracy

**Figure 3:**
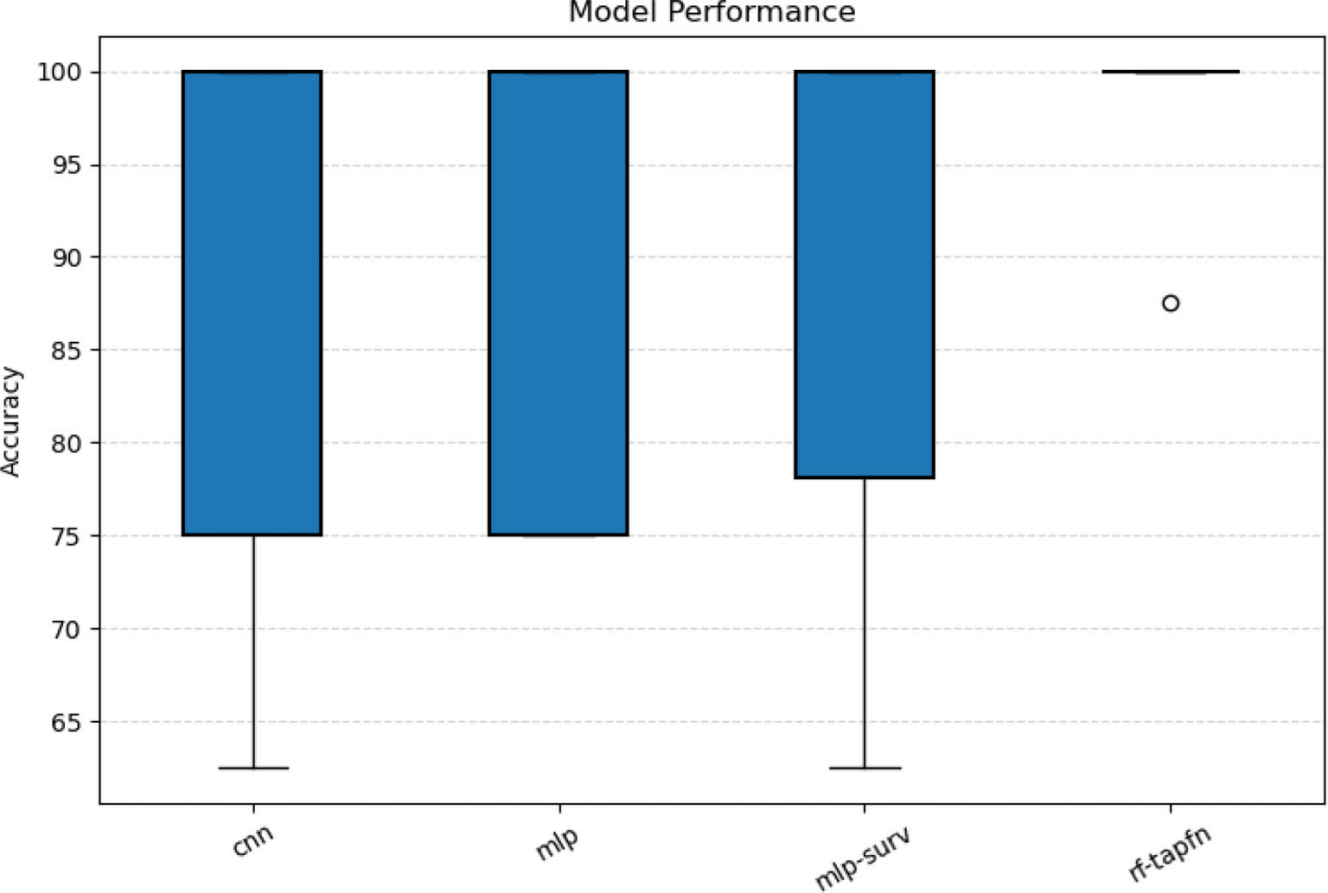
The figure compares the performance distribution of different baseline models on the PSI-MS dataset. For all models, we display the performance together with the best compression method used. Standard deep learning techniques perform best when combined with AE-based compression, while TabPFN achieves its highest performance with tree-based feature pre-selection. For all models, we perform hyperparameter optimization over the number of input features, starting with a minimum of 40 features and increasing in steps of 20 up to a maximum of 500 features. Shown are the performance of every outer CV.

**Figure 4:**
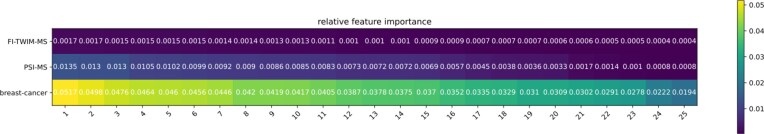
The heatmap illustrates the relative significance of the top 25 features from both datasets, as determined by scaled SHAP (Shapley Additive Explanations) values. Each cell represents the contribution of a feature to the model’s prediction, with values on the left denoting greater importance. In the FI-TWIM-MS dataset, the importance of features is more evenly distributed among the top 20 values. Conversely, the PSI-MS dataset exhibits a more concentrated distribution of feature importance across various variables, indicating a broader and more diffuse biomarker signature. The BC dataset shows particularly high values for the top 25 values, explaining its relative stability in performance regardless of feature selection.

We systematically varied the feature count using a RF-based selection approach to evaluate the relationship between the number of selected features and classification accuracy. For the FI-TWIM-MS dataset, the results reveal that using only 80 features achieves near-optimal accuracy. Beyond 150 features, the accuracy plateaus and further increases in feature count introduce a mild risk of overfitting, particularly given the small sample size. This suggests that a smaller subset of highly informative features is sufficient to distinguish prostate and BC cases from healthy controls. In contrast, the PSI-MS dataset exhibits a more pronounced dependency on feature count, with significant improvements in accuracy observed as the number of features increases. The hyperparameter optimization returns similar sizes of feature selection for all folds. Figure 3. shows the accuracy distribution for all 10 folds on the optimized architectures. In Figure 4, we analyze the distribution of importance among the 20 most important features in each dataset. For the FI-TWIM-MS dataset, we find that the importance is more evenly distributed, while for the PSI-MS dataset, the importance is more concentrated.

These findings highlight the importance of dataset-specific feature selection strategies. For the FI-TWIM-MS dataset, recombining existing features in the latent space yields the best results. For the higher-dimensional PSI-MS dataset, we found that pre-selecting the most valuable features, which have the most significant impact on performance, is more effective. This highlights the versatility of the proposed approach in optimizing performance across diverse datasets.

### Comparing the proposed model to other methods

To assess the effectiveness of the proposed AE/RF–TabPFN pipeline, we performed a benchmarking analysis against various deep learning and machine learning baseline models, using identical preprocessing and feature normalization procedures. A nested 10-fold stratified CV approach was employed to ensure fair comparison among the models and to support robust hyperparameter optimization.

1. Performance on prostate datasets: In the analysis of the FI-TWIM-MS serum dataset, the proposed methodology achieved a ROC-AUC of 0.96 and an F1-score of 0.89 when employing tree-based feature pre-selection. This performance slightly surpasses that of the AE-based compression variant, which recorded an ROC-AUC of 0.95 and an F1-score of 0.84. For the PSI-MS urine dataset, the same configuration attained an impressive ROC-AUC of 0.99, together with an F1-score of 0.99. These results highlight the robustness of the method across different ionization modalities.
2. Performance on the breast-cancer dataset: In the analysis of the MiniMaP breast-biopsy PSI-MS dataset, the AE-based classifier achieved a ROC-AUC of 0.92, with an F1 score of 0.89. In comparison, the variant using RF feature selection showed a marginal improvement, reaching an ROC-AUC of 0.93 and an F1 score of 0.90. These results support the model’s ability to generalize effectively across different PSI-MS domains.
3. Baseline model comparison: Alternative deep learning baselines, including a compact one-dimensional convolutional neural network (1D-CNN), a MLP, and an MLP-Surv architecture, were evaluated under consistent CV conditions.
4. The 1D-CNN demonstrated impressive performance, achieving ROC-AUC scores of 0.94 (F1 score 0.87) on the FI-TWIM-MS dataset, 0.93 (F1 score 0.89) on the PSI-MS dataset, and 0.92 (F1 score 0.90) on the breast dataset. In comparison, the standard MLP attained ROC and F1 scores of 0.95 and 0.88, respectively, on the FI-TWIM-MS dataset, and 0.95 and 0.82 on the PSI-MS dataset when using tree-based selection methods. Alternatively, using AE-based compression, the MLP achieved ROC and F1 scores of 0.919 and 0.90, and 0.925 and 0.86 for the respective datasets. On the breast dataset, AE-based compression resulted in ROC and F1 scores of 0.92 and 0.90. The MLP-Surv model showed competitive yet marginally lower performance, with ROC-AUC scores of 0.94 (F1 score 0.87) on the FI-TWIM-MS dataset, 0.93 and 0.86 on the PSI-MS dataset, and 0.92 and 0.88 on the breast dataset, using tree-based pre-selection methods. Notably, the AE-based preprocessing of features tended to outperform the tree-based pre-selection on the baseline methods.
5. The XGBoost classifier, using its built-in feature selection and evaluated through 10-fold CV, achieved ROC-AUC and F1 score pairs of 0.91/0.83 for FI-TWIM-MS, 0.96/0.89 for PSI-MS, and 0.91/0.88 for BC. Although XGBoost showed competitive performance, its metrics were consistently lower than those obtained with the proposed TabPFN-based pipeline.
6. Interpretation and scalability: TabPFN consistently demonstrated superior performance compared to traditional models across various datasets by effectively utilizing prior-data fine-tuning and probabilistic feature reasoning, particularly in scenarios characterized by small sample sizes and high-dimensionality, which are typical in metabolomics. Unlike conventional machine learning models such as RF and XGBoost, which depend heavily on explicit feature engineering, TabPFN incorporates domain-agnostic priors and learns non-linear dependencies autonomously.
7. This advantage becomes increasingly evident as dataset complexity escalates, emphasizing the scalability of deep foundation-model architectures for applications in metabolomics diagnostics. Figure 4 presents a summary of the comparative performance across all models and datasets, illustrating the superior distributions of ROC-AUC and F1-score achieved by the proposed AE/RF–TabPFN pipeline.

### Classification by disease condition and experimental type

In the next step of the analysis, the performance of our model was verified using a consolidated dataset that combined the urine-based PSI-MS dataset and the serum-based FI-TWIM-MS dataset. To establish a unified feature space, we initially employed a RF classifier to select the top 140 most significant features (ranked by their contribution to classification efficiency). The two datasets were then merged and normalized prior to the training and evaluation of the model.

**Figure 5:**
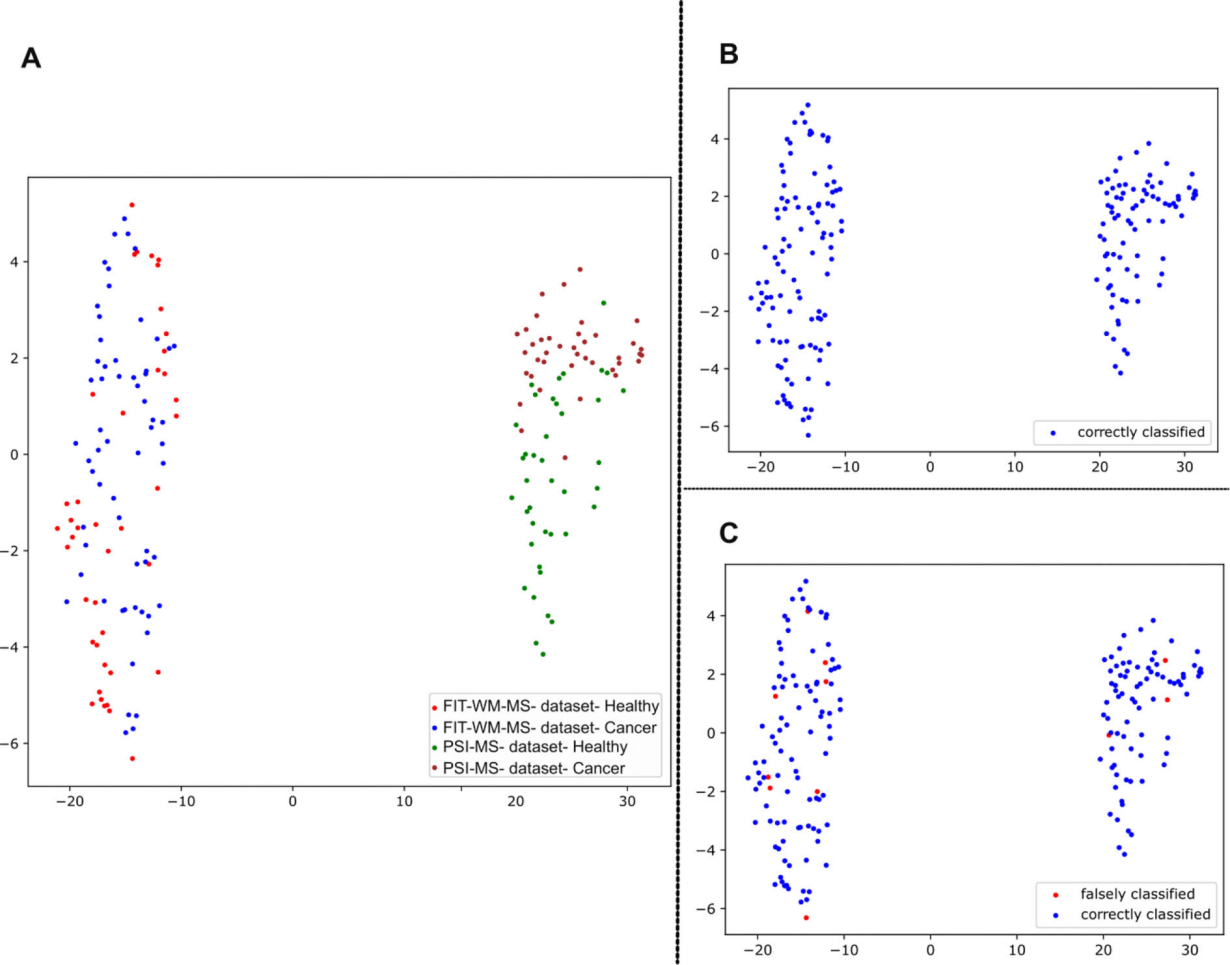
t-SNE plot of combined samples for both datasets used for the classification with AE-TabPFN on four classes (two datasets × two conditions). Panel (a) shows the ground truth for 2 datasets and 2 conditions. Panel (b) shows the classification results only regarding classification in dataset 1 or dataset 2. We find that our approach can distinguish between both methods with 100% accuracy. Panel (c) shows the correctly and falsely classified samples of both datasets according to the ground truth shown in (a).

As illustrated in Fig. 5, the t-SNE projection of the resulting dataset reveals that samples predominantly cluster according to the analytical technique employed, rather than by clinical condition. This makes it more challenging to differentiate between healthy and diseased individuals than to distinguish between PSI-MS and FI-TWIM-MS sources.

Despite this challenge, the resulting RF–TabPFN-140 model demonstrated robust predictive performance, achieving an accuracy of 94%, a weighted F1-score of 0.94 and an ROC-AUC of 0.99.

Figure 5a presents the original two-dimensional t-SNE representation of the integrated feature space. Figure 5b highlights the samples correctly classified as belonging to the first or second dataset. We achieve a virtual 100% accuracy. Figure 5c illustrates classification outcomes based on both dataset origin and clinical condition. It is noteworthy that the model consistently classified samples accurately according to their dataset of origin, while classification based on health status proved more challenging, likely due to cross-platform variability. This highlights the need for improvements in the integration of different types of datasets to make use of a more unified approach. Due to the difference in the feature space additional batch correction methods would currently not be appropriate.

### Examining the spectral data across datasets

**Figure 6:**
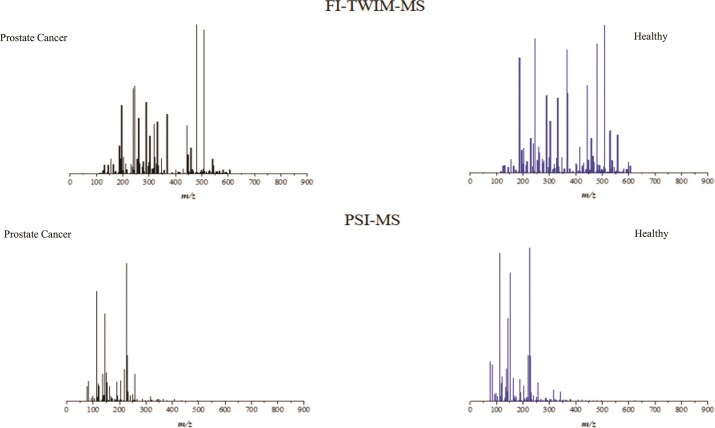
It presents the group-averaged metabolomic spectral profiles obtained from healthy controls and PC patients utilizing two advanced MS techniques. PSI-MS and FI-TWIM-MS. Although both techniques encompass the same m/z range on the *x*-axis, their spectral profiles reveal distinct characteristics. PSI-MS and FI-TWIM-MS demonstrate notable differences in peak intensities and distributions.

To further evaluate the challenges of cancer diagnosis using metabolomic data, we analyzed the group-averaged spectral profiles of diseased and healthy individuals for both datasets from both PSI-MS and FI-TWIM-MS. Figure 6 showcases these profiles, as well as both datasets. The raw mass spectra shared similar m/z ranges; however, subtle variations in intensity and peak distribution were observed.

The distinctions observed were not immediately apparent through conventional spectral inspection due to the complex nature and high dimensionality of the metabolomic profiles. It is essential to note that, although the mass ranges assessed were consistent, the spectral profiles generated by the two techniques displayed variations in resolution, baseline noise, and peak sharpness. These differences reflect fundamental discrepancies in ionization mechanisms as well as in the sample matrices employed.

The model addresses this challenge by identifying non-linear relationships and utilizing subtle differences in the spectral data to enhance classification accuracy. For both datasets, the spectral profiles of diseased and healthy individuals overlap even more extensively. The overlapping nature of these profiles makes it nearly impossible for manual or linear approaches to differentiate between the two groups effectively. Nonetheless, the proposed model achieves high accuracy, demonstrating its robustness in handling challenging datasets.

These findings highlight the importance of integrating deep learning with spectral analysis. The AE-TabPFN model excels at processing complex, noisy datasets, enabling reliable classification even when spectral profiles are visually indistinguishable. This capability is crucial for advancing metabolomics-based diagnostics, where the accurate interpretation of subtle spectral differences can significantly enhance disease detection and monitoring.

## Conclusion

This study presents a novel diagnostic method that integrates an AE for dimensionality reduction, feature pre-selection via RFs and SHAP analysis, and a TabPFN classifier to analyze high-dimensional metabolomic data derived from PSI-MS (urine) and FI-TWIM-MS (serum) sources. This work provides a comprehensive benchmark for heterogeneous metabolomics datasets from different analytical platforms (PSI-MS, FI-TWIM-MS, and MiniMaP PSI-MS) and various biological matrices (urine, serum, and tissue) for both prostate and BC. The proposed approach demonstrated exceptional diagnostic efficacy in detecting PC, achieving classification accuracies of up to 98.75%, despite the inherent complexity and limited size of the datasets. While these results are so far very promising, it is not yet entirely clear whether the strong performance on the BC dataset depends on the strong signal present in the biopsy dataset.

The integration of explainable AI methodologies with deep learning has facilitated robust predictive capabilities alongside biological interpretability. Interestingly, significant PC-related biomarkers, including creatinine and TMAO (trimethylamine N-oxide), were consistently identified, thereby reinforcing the model’s biological relevance. The AE component adeptly minimized noise in high-dimensional input while retaining essential latent features. Additionally, the pre-trained architecture of TabPFN ensured effective generalization, even in contexts of limited sample sizes.

This work highlights the potential of rapid, non-invasive diagnostic techniques employing ambient MS methods, specifically PSI-MS and FI-TWIM-MS, in conjunction with advanced AI models that can process heterogeneous and high-dimensional datasets. In contrast to conventional workflows that necessitate extensive sample preparation and lengthy chromatographic processes, the proposed approach demonstrates efficiency with minimal preprocessing, making it suitable for real-time applications in clinical settings.

Despite the promising results obtained, further validation through multi-institutional and longitudinal datasets is essential to establish reproducibility, particularly across various disease stages and diverse patient populations. Additionally, standardizing sample collection protocols, along with integrating clinical metadata, remains a critical step toward the routine implementation of these findings in clinical practice.

In summary, the AE/RF-TabPFN approach offers a scalable and interpretable approach for cancer diagnostics based on metabolomics. This framework establishes a promising baseline from which future work can build clinical approaches for classification in precision medicine, not only within the field of oncology but also potentially across various metabolic disorders, by providing a more standardized process integrated with clinical metadata.

## Availability of source code and requirements

Project name: Autoencoder/RandomForest–TabPFN

project home page: https://github.com/SvenHauns/metabolemic_classifier.

Operating system: Platform independent.

Programming language: Python.

Other requirements: To install software requirements, we provide a Conda environment file in the GitHub repository.

License: MIT License.

## Supplementary Material

giag053_Authors_Response_To_Reviewer_Comments_original_submission

giag053_GIGA-D-25-00458_original_submission

giag053_GIGA-D-25-00458_revision_1

giag053_Reviewer_1_Report_original_submission Reviewer 1 -- 12/4/2025

giag053_Reviewer_1_Report_revision_1 Reviewer 1 -- 4/14/2026

giag053_Reviewer_2_Report_original_submission Reviewer 2 -- 1/11/2026

giag053_Reviewer_2_Report_revision_1 Reviewer 2 -- 4/16/2026

## Data Availability

The model used is available under Github repository [26]. All additional supporting data are available in the GigaScience repository [27], GigaDB [28].
