# Supplementary material for: Autoencoder/RandomForest–TabPFN for cross-cancer metabolomics: prostate and breast cancer diagnosis using paper spray and ion mobility-mass spectrometry techniques
Source: Gigascience. 2026 May 7;15:giag053. doi: 10.1093/gigascience/giag053 (PMC13251742; doi:10.1093/gigascience/giag053)
Supplement: giag053_GIGA-D-25-00458_original_submission [file giag053_giga-d-25-00458_original_submission.pdf]

GigaScience

Autoencoder/RF–TabPFN for Cross-Cancer Metabolomics: Prostate and Breast Cancer Diagnosis Using PSI-MS and FI-TWIM-MS

--Manuscript Draft--

**Manuscript Number:**	GIGA-D-25-00458
**Full Title:**	Autoencoder/RF–TabPFN for Cross-Cancer Metabolomics: Prostate and Breast Cancer Diagnosis Using PSI-MS and FI-TWIM-MS
**Article Type:**	Technical Note
**Funding Information:**	Mohammed Bin Rashid University of Medicine and Health Sciences (MBRU-CM-RG2024-07) — Dr Omer Alkhnbashi<br>Mohammed Bin Rashid University of Medicine and Health Sciences (MBRU-CM-RG2025-12) — Dr Omer Alkhnbashi
**Abstract:**	Accurate and rapid disease diagnosis, particularly in prostate cancer (PC), is critical for early intervention and improved patient outcomes. Metabolomic signatures represent a robust molecular framework for elucidating cancer-associated biochemical reprogramming. The use of Artificial Intelligence (AI) in biology in recent years has become widespread and promising. This study introduces a novel predictive method that integrates an Autoencoder, random forest-based feature selection and Tabular Prior-data Fitted Network (TabPFN) to achieve high diagnostic accuracy from metabolomics data of prostate cancer patients. The datasets were acquired using Paper Spray Ionization Mass Spectrometry (PSI-MS) and Flow Injection–Two-Way Mirror Mass Spectrometry (FI-TWIM-MS) of individuals diagnosed with PC. Leveraging metabolomic profiling data from two distinct sources prostate cancer urine and serum samples, the proposed model achieved an accuracy up to 98.75% in distinguishing diseased from healthy condition. Additionally, we employed a breast cancer dataset containing metabolic and lipidomic signatures acquired from core needle biopsies using a miniature MS platform coupled with PSI to assess the fidelity of our implementation across distinct cancer types. Our results on a well-characterized targeted dataset shows that we can effectively reduce high-dimensional data into latent feature representations. At the same time, TabPFN captures tumor progression-related changes and models temporal dependencies, thereby enhancing the possibility that the model will be a highly potent and effective tool for stage-specific diagnostic precision.<br>Most existing machine learning approaches for disease diagnosis primarily rely on imaging, genomics, or clinical parameters, often overlooking the critical role of metabolites in identifying disease-specific biochemical signatures. By integrating metabolite-specific data with a robust deep-learning approach, this study demonstrates the transformative potential of AI in metabolomics-based diagnostics. The proposed model offers scalability and versatility, with applications extending beyond oncology to a much broader disease profiling aspect. These findings emphasise the value of combining multi-source metabolomic data with deep learning to advance personalised medicine and enhance diagnostic efficiency in clinical practice.
**Corresponding Author:**	Omer Alkhnbashi, PhD<br>MBRU: Mohammed Bin Rashid University of Medicine and Health Sciences<br>Dubai, UNITED ARAB EMIRATES
**Corresponding Author Secondary Information:**	
**Corresponding Author's Institution:**	MBRU: Mohammed Bin Rashid University of Medicine and Health Sciences
**Corresponding Author's Secondary Institution:**	
**First Author:**	Omer Alkhnbashi, PhD
**First Author Secondary Information:**	
**Order of Authors:**	Omer Alkhnbashi, PhD

Powered by Editorial Manager® and ProduXion Manager® from Aries Systems Corporation

	Sven Hauns
	Frederico G. Pinto
	Costerwell Khyriem
	Ankita Singh
	Azzat Al-Sadi
	Talal Al Yazeed
	Rasheed Mohammad
	Babacar Cisse
	Timothy J. Garrett
	Mohammed Uddin
	Nelson C. Soares
	Rolf Backofen
**Order of Authors Secondary Information:**	
**Additional Information:**	
**Question**	**Response**
Are you submitting this manuscript to a special series or article collection?	No
**Experimental design and statistics**<br><br>Full details of the experimental design and statistical methods used should be given in the Methods section, as detailed in our Minimum Standards Reporting Checklist. Information essential to interpreting the data presented should be made available in the figure legends.<br><br>Have you included all the information requested in your manuscript?	Yes
**Resources**<br><br>A description of all resources used, including antibodies, cell lines, animals and software tools, with enough information to allow them to be uniquely identified, should be included in the Methods section. Authors are strongly encouraged to cite Research Resource Identifiers (RRIDs) for antibodies, model organisms and tools, where possible.	Yes

Have you included the information requested as detailed in our Minimum Standards Reporting Checklist?	
**Availability of data and materials**<br><br>All datasets and code on which the conclusions of the paper rely must be either included in your submission or deposited in publicly available repositories (where available and ethically appropriate), referencing such data using a unique identifier in the references and in the “Availability of Data and Materials” section of your manuscript.<br><br>Have you have met the above requirement as detailed in our Minimum Standards Reporting Checklist?	Yes
GigaScience has policies and guidelines in place for the use of generative AI-writing tools such as ChatGPT. If you have used such writing tools to assist with writing the manuscript this must be declared and cited in the text. Authors should not list AI-writing tools and other AI-assisted technologies as an author or co-author and should acknowledge that they are fully responsible for text generated or refined by AI-writing tools.<p><br><br>A summary of use (particularly in the introduction or among methods) needs to be included at the end of the paper, and the outputs should also be included as a supplementary file hosted in GigaDB or other open repositories. Please read our guidelines for more information. <p><br><br>By submitting to GigaScience, you are aware of the journal's AI-writing tools policy, and if you have declared use of such tools below, you have acknowledged this where appropriate in your manuscript and have made a summary of use and outputs available. <p><br><b>AI-assisted writing tools have been used in the preparation of this manuscript?	No

Powered by Editorial Manager® and ProduXion Manager® from Aries Systems Corporation

Manuscript

Click here to access/download;Manuscript;Manuscript_Deeplearning-

Autoencoder/RF–TabPFN for Cross-Cancer Metabolomics: Prostate and Breast Cancer Diagnosis Using PSI-MS and FI-TWIM-MS

Sven Hauns[1], Frederico G. Pinto[2,3], Costerwell Khyriem[4], Ankita Singh[2], Azzat Al-Sadi[5], Talal Al Yazeedi[2], Rasheed Mohammad[6], Babacar Cisse[7], Timothy J. Garrett[8,9], Mohammed Uddin[2,4,10], Nelson C. Soares[2,4,11,12], Rolf Backofen[1,13,*], Omer S. Alkhnbashi[2,4,*]

[1]Bioinformatics group, Department of Computer Science, University of Freiburg, 79085 Freiburg, Germany
[2]Center for Applied and Translational Genomics (CATG), Mohammed Bin Rashid University of Medicine and Health Sciences, Dubai Health, Dubai P.O. Box 505055, United Arab Emirates.
[3]Institute of Exact Sciences, Federal University of Viçosa, Rio Paranaíba 38810-000, Brazil
[4]College of Medicine, Mohammed Bin Rashid University of Medicine and Health Sciences, Dubai Health, Dubai P.O. Box 505055, United Arab Emirates
[5]Department of Computer Engineering, Hadhramout University, Hadhramout, Yemen
of Computer Sciences, College of and Digital Technology, Birmingham City University, Birmingham B4 7XG, UK
[7]Department of Neuroscience, Dubai Health, Dubai P.O. Box 505055, United Arab Emirates.
[8]Department of Pathology, Immunology, and Laboratory Medicine, University of Florida, Gainesville, Florida 32610, United States
[9]Southeast Center for Integrated Metabolomics, Clinical and Translational Science Institute, University of Florida, Gainesville, Florida 32610, United States
[10]GenomeArc Inc, Mississauga, Ontario, Canada
[11] Laboratory of Proteomics, Department of Human Genetics, National Institute of Health Doutor Ricardo Jorne (INSA), Lisbon, Portugal
[12]Comprehensive Health Research Centre (CHRC), NOVA Medical School, University NOVA of Lisbon, Lisbon, Portugal
[13]Signalling Research Centres BIOSS and CIBSS, University of Freiburg, 79085 Freiburg, Germany

Abstract

Accurate and rapid disease diagnosis, particularly in prostate cancer (PC), is critical for early intervention and improved patient outcomes. Metabolomic signatures represent a robust molecular framework for elucidating cancer-associated biochemical reprogramming. The use of Artificial Intelligence (AI) in biology in recent years has become widespread and promising. This study introduces a novel predictive method that integrates an Autoencoder, random forest-based feature selection and Tabular Prior-data Fitted Network (TabPFN) to achieve high diagnostic accuracy from metabolomics data of prostate cancer patients. The datasets were acquired using Paper Spray Ionization Mass Spectrometry (PSI-MS) and Flow Injection–Two-Way Mirror Mass Spectrometry (FI-TWIM-MS) of individuals diagnosed with PC. Leveraging metabolomic profiling data from two distinct sources prostate cancer urine and serum samples, the proposed model achieved an accuracy up to 98.75% in distinguishing diseased from healthy condition. Additionally, we employed a breast cancer dataset containing metabolic and lipidomic signatures acquired from core needle biopsies using a miniature MS platform coupled with PSI to assess the fidelity of our implementation across distinct cancer types. Our results on a well-characterized targeted dataset shows that we can effectively reduce high-dimensional data into latent feature representations. At the same time, TabPFN captures tumor progression-related changes and models temporal dependencies, thereby enhancing the possibility that the model will be a highly potent and effective tool for stage-specific diagnostic precision.

Most existing machine learning approaches for disease diagnosis primarily rely on imaging, genomics, or clinical parameters, often overlooking the critical role of metabolites in identifying disease-specific biochemical signatures. By integrating metabolite-specific data with a robust deep-learning approach, this study demonstrates the transformative potential of AI in metabolomics-based diagnostics. The proposed model offers scalability and versatility, with applications extending beyond oncology to a much broader disease profiling aspect. These findings emphasise the value of combining multi-source metabolomic data with deep learning to advance personalised medicine and enhance diagnostic efficiency in clinical practice.

Introduction

The accurate and timely diagnosis of diseases, particularly cancers, is crucial for enhancing patient outcomes and facilitating early intervention. Although traditional diagnostic approaches—such as histopathological examinations, imaging modalities, and biochemical assays—remain the cornerstone of definitive disease diagnosis, they are often invasive, time-consuming, and costly. Moreover, these methods may lack the sensitivity and specificity necessary for early detection or for distinguishing between disease subtypes. This underscores the need for innovative, less-invasive diagnostic approaches that combine advanced molecular profiling techniques with robust computational frameworks to transform disease detection and management [1,2].

Metabolomics, i.e. the comprehensive capture and analysis of small molecules in biological systems, has emerged as a powerful tool in modern diagnostics. Leveraging high throughput mass spectrometry (MS) platforms, such as paper spray ionization mass spectrometry (PSI-MS) and flow injection−traveling-wave ion mobility−mass spectrometry (FI-TWIM-MS), metabolomics enables the identification of key biomarkers associated with disease states, offering insights into underlying molecular mechanisms.

Conventional metabolomic workflows, particularly those based on liquid chromatography coupled with mass spectrometry (LC-MS), often necessitate comprehensive sample preparation and protracted chromatographic separation. These constraints limit their applicability in clinical settings, where expeditiousness and simplicity are paramount. In contrast, the emerging techniques of FI-TWIM-MS and PSI-MS represent rapid and cost-efficient high-throughput alternatives that eliminate the need for complex and time-consuming chromatographic separation while maintaining analytical sensitivity. PSI-MS facilitates direct analysis from dried biofluid spots within seconds, while FI-TWIM-MS integrates rapid injection with gas-phase ion mobility to enhance resolution and mitigate spectral congestion. Collectively, these methodologies substantially diminish analysis time and streamline workflows, positioning them as promising instruments for real-time, point-of-care disease diagnostics.

PSI-MS-based metabolomics has gained traction for rapidly analyzing biofluids like urine, blood, and serum without complex sample preparation [3,4,5]. For example, PSI-MS has been used to identify distinct metabolic signatures in prostate and breast cancers, achieving good diagnosis by integrating advanced chemometric and traditional analyses [6,7,8]. In PSI-MS, the sample is deposited onto a triangular piece of paper that is connected to a high-voltage source and positioned near the inlet of the mass spectrometer. When a solvent is introduced and high voltage (typically 3 to 5 kV) is applied, charged droplets are generated at the tip of the paper triangle. This process extracts analytes from the sample using the solvent and transports them to the tip through capillary action. The application of high voltage leads to the formation of a Taylor cone at the paper's tip, similar to the process observed in electrospray ionization (ESI), resulting in the generation of gas-phase ions that are detected by the mass spectrometer [9]. This technique demonstrates considerable potential for reduced cost- and labor-efficient as well as fast disease diagnosis. Flow Injection - Traveling Wave Ion Mobility - Mass Spectrometry (FI-TWIM-MS) is an analytical technique that combines the rapid, chromatography-free sample introduction of Flow Injection (FI) with the gas-phase ion separation capabilities of Traveling Wave Ion Mobility (TWIM) and high-resolution mass analysis of Mass Spectrometry (HRMS). In FI, the sample is directly introduced into the mass spectrometer via a continuous solvent flow, bypassing the time-consuming chromatographic separation step. Subsequently, ions are separated in the TWIM cell based on their size, shape, and charge as they travel through a gas under the influence of a traveling wave electric field. Finally, the separated ions are analyzed by the mass spectrometer, providing information about their mass-to-charge ratio. This hyphenated technique offers a rapid and efficient approach for complex mixture analysis, reducing spectral congestion and enabling the acquisition of collision cross-section (CCS) values, which provide additional structural information about the analytes [10]. FI-TWIM-MS was applied to discriminate between healthy and prostate cancer patients using serum samples [10]. Unlike PSI-MS where the sample preparation step can be eliminated depending on the sample matrix, using FI-TWIM-MS the sample needs to be prepared prior to analysis.

Despite these technological advancements, metabolomic data remains high-dimensional and subject to noise, necessitating sophisticated analytical approaches to extract salient patterns [11,12]. Artificial intelligence (AI) and intensive learning techniques present robust solutions to this challenge. Autoencoders compress input features into latent representations, effectively capturing complex interrelations within the data. Furthermore, transformer-based models such as TabPFN [13], which are pre-trained on extensive tabular datasets, facilitate robust classification even when sample sizes are limited [9,10].

The integration of AI with rapid mass spectrometry technologies fosters the development of scalable, real-time, and noninvasive diagnostic frameworks. However, challenges persist

concerning the standardization of protocols, the management of heterogeneous data sources, and the achievement of clinical scalability [11,12,13,14,15].

To address these challenges, we propose an innovative approach that combines autoencoder-based compression, feature selection based on random forests, and, as a final step, the TabPFN classifier to analyze metabolomic data from two prostate cancer studies; one employing PSI-MS (urine) [6,7] and the other utilizing FI-TWIM-MS (serum) [10]. Additionally, we included one breast cancer metabolic profile based on paper-spray ionization miniature mass spectrometry (PSI-MS) data to examine the utility of TabPFN on a disease similar to PC but completely unrelated [16]. This multi-source, AI-integrated approach has achieved classification accuracy of up to 98.75%, underscoring its potential to advance personalized medicine and enhance clinical decision-making in the field of oncology.

Method

Datasets

In this study, we utilize three different datasets comprising metabolomic data. The first two datasets are on prostate cancer (PC) metabolomics. The first dataset was derived from a clinical metabolomics study that employed Flow Injection–Traveling-Wave Ion Mobility Mass Spectrometry (FI-TWIM-MS) to analyze serum samples obtained from 61 prostate cancer patients and 42 healthy controls (total of 103 samples) [10] and 237 metabolite features per sample. The serum samples underwent processing through a cold solvent extraction protocol, followed by phase separation to eliminate lipids and proteins. FI-TWIM-MS facilitated rapid analysis, with each sample being processed in approximately six minutes, including wash runs, while concurrently separating ions based on both mass-to-charge ratio (m/z) and collision cross section (CCS). The use of ion mobility enhanced resolution within complex mixtures contributed high-quality input for downstream classification tasks. The second dataset consists of urine-based metabolic profiles, which were acquired using Paper Spray Ionization Mass Spectrometry (PSI-MS). This data was sourced from previously published studies [6,7] that involved urine samples obtained from 40 prostate cancer patients, as well as from 40 healthy control subjects. The PSI-MS technique facilitated rapid and direct ionization of dried urine spots with minimal sample preparation, yielding comprehensive mass spectra across the m/z range. After filtering the dataset contained 784 metabolite features per sample. These features represent intensity values at different m/z. In contrast, the third dataset utilized paper-spray ionization miniature mass spectrometry (PSI-MS) to analyse core-needle breast biopsies [16]. It included 692 samples, with 204 malignant and 488 benign cases, each characterized by 494 metabolite features, obtained using a miniature MS platform coupled with PSI, the MiniMaP platform. The spectra, covering an m/z range of 500 to 1000, mainly reflected lipid profiles annotated by histopathological examination. This dataset was used to

evaluate the cross-disease and cross-platform generalizability of the Autoencoder/RF–TabPFN approach. All datasets were pre-processed using L_2-normalization to standardize feature scales before model training

General Strategy for Model Selection

To evaluate the quality of our predictions, we utilize nested 10-fold CrossValidation to ensure a fair and robust performance evaluation for our hyperparameter optimization. The final models are then created by averaging the hyperparameter result of the nested CV (latent dimension or number of selected features) and then retraining the model on the data used for the inner CV, while evaluating it on the outer CV to guarantee an independent test set.

Since metabolomic datasets often exhibit high dimensionality but contain relatively few samples, we explore two techniques to select informative input features and mitigate the risk of overfitting. These techniques also help focusing on the most salient input features, while disregarding redundant information in the temporally structured metabolomic data, and improve the classification performance Figure 1 provides an overview of this feature selection pipeline, showing the two main strategies: Autoencoder-based latent feature extraction and Random Forest-based feature importance estimation. Those two strategies were used prior to final classification with the TabPFN model.

Our model was applied as the first step of the feature reduction approach to provide TabPFN with a smaller set of important features. For this first step, we use two different methods. The first is a simple feature selection process based on Shannon entropy and information gain, calculated from random forest splits. Here, Shannon entropy is used at each node of a tree to determine how well a potential feature splits the data into distinct groups, measuring the uncertainty within each subset of samples. A split that maximizes information gain and hence minimizes entropy is chosen. For trees created in this manner, feature importance can be calculated by summing the total information gained by that feature across all data splits and all trees. This approach enables identifying a variable number of highly informative input features and assess how the final prediction accuracy changes with different numbers of selected features.

The second method employs an autoencoder, where the latent space serves as the feature extraction layer for subsequent classification tasks. A latent embedding was used as a compressed representation of the input features and later to analyse the importance of specific input features. To ensure the autoencoder effectively captures relevant information, we condition it on both input reconstruction and final prediction by combining these objectives into a single loss function. Additionally, Shapley values were employed to identify which input features have the greatest impact on constructing the autoencoder's latent space.

The final classification is then undertaken to train the TabPFN [13] model on the preselected optimal features. Due to being pre-trained on input data described by up to 500 features, our

feature reduction methods effectively transform the original input space to a dimension TabPFN can make best use of. Furthermore, it allows to assess feature importance and thus explainability, which is critical for acceptance of the method by medical professionals.

Architecture and training

Since the data is very sparse, we rely on a pre-trained model to enable reliable classification while avoiding overfitting. We combine feature selection, based on random forest feature importance, and feature compression using autoencoders with TabPFN for optimal classification results. The performance is evaluated by first fitting TabPFN to the data on the training fold and predicting the output on the test folds. In addition, to gain more insight into the structure of the feature space, we also applied a random forest-based approach for direct feature selection for classification and to gain insight into the importance of the input features. To overcome the possibility of overfit, sampling was applied to ensure an even distribution of positive and negative samples in every fold.To ensure a fair evaluation of our method, all hyperparameter optimizations were run on a nested 10-fold Cross-Validation. The returned hyperparameter were averaged and the final model retrained and evaluated on the test sets of the outer CV split. All baseline methods were trained for 100 epochs using an Adam optimizer and evaluated using 10-fold nested CV when we make use of hyperparameter optimization.

Comparison to baseline

We employ multiple baseline methods to compare the combination of TabPFN with compression and feature selection against a selection of deep learning methods trained using the same compression pipeline. The first baseline model is a 1D CNN that expands the input channel dimension to 128 through three convolutional layers with kernel sizes of 2, followed by average pooling. This design allows us to effectively utilize small feature-selection sets and compressed representations. The second baseline model is a basic MLP consisting of three layers with ReLU activation functions and batch normalization. The third deep learning baseline model, which we refer to as MLP-surv due to its resemblance to the general architecture of DeepSurv [17], comprises three layers with alternating linear layers, batch normalization, and dropout.

Input feature importance

To enhance model interpretability and improve feature selection, two complementary approaches were applied: random forest-based feature selection and autoencoder-driven

latent space analysis (as seen in Fig 2). Firstly, random forests were utilized to rank input features according to their importance in classification. Feature importance was determined using Shannon entropy and information gain, calculated from random forest splits. This enables assessing the effects of different feature subsets on classification accuracy by selecting several; and the most salient; features. An autoencoder-based latent space was incorporated to extract meaningful representations from high-dimensional metabolomic data. The autoencoder was trained to reconstruct input data, while concurrently optimising for classification performance. To interpret which input features contributed most significantly to the latent space, SHAP values were applied, which quantify the impact of each feature on the encoded representation. By combining these two methods, the aim was to identify the most relevant biomarkers, while minimising noise and redundancy in the dataset. This feature selection strategy was implemented before training the TabPFN classifier to ensure that the most informative variables-only- contributed to the model predictions. Unlike random forest classifiers, autoencoders allow us to create a latent space from the entire input sequence. The model automatically learns a representation that captures all vital information for the final classification.

Results and Discussion

Our Deep-Learning Model achieves high accuracy on a diverse set of tasks.

For both datasets, we test different feature pre-processing methods to assess the best way of dealing with high-dimensional data with a low number of samples. The first is autoencoder-based, where the input feature vectors are mapped to a latent space with a lower dimension, and the features of the latent space are used as input for TabPFN. The second is based on direct feature selection, which is performed using the estimation of feature importance by a random forest classifier (see Methods). We thus term the different models AE-TabPFN-FS or RF-TabPFN-FS, where the first part determines the used method for feature selection (AE=autoencoder, RF=random forest), and FS describes the final TabPFN input feature size. For the FI-TWIM-MS dataset RF-TabPFN-210 results in an accuracy of 91%, with a weighted F1-score of 0.91 and a ROC-AUC of 0.95. For the PSI-MS dataset, pre-selecting features using a random forest and then fine tuning TabPFN (RF-TabPFN-236) creates an accuracy of 98.75%, a weighted F1-score of 0.99 and a ROC-AUC of 1.0. Though only a smaller number of features were removed, it still outperforms TabPFN on the complete feature set, so combining TabPFN with feature selection is superior in our type of data (see Table 1). For the breast cancer dataset [16] using the most important 170 features RF-TabPFN-170 reaches

an accuracy of 90 % with an ROC-AUC of 0.94 and F1-score of 0.90. Similar results are achieved with AE-TabPFN-240 with an accuracy of 89 % and an ROC-AUC of 0.92.

Finally, we compare our method with standard machine learning methods on the same data split and find an improvement in our method over the alternatives. For the complete evaluation, see Figure 4.

Insights from the determined feature importance

We utilised SHAP (Shapley Additive Explanations) values for two datasets to identify the most critical input features. Figure 2 highlights the distribution of feature importance across these datasets. In the FI-TWIM-MS dataset, which comprises prostate cancer urine samples. The most predictive features were predominantly located within the middle m/z range. This particular region is likely to include lipid-based metabolites such as phosphatidylcholines and triacylglycerols. These two molecules have been previously reported to be indicative of cancer metabolic reprogramming [18, 19, 20], pro-tumorigenic signalling [22, 23], resistance [24, 25], and metastasis [26, 27]. Particularly in prostate cancer, elevated phosphatidylcholines in exosomes from hormone-sensitive PCa cell lines LNCaP correlate with aggressive disease [PMID: 33854590]. Conversely, lysophosphatidylcholines (LPCs) in blood are linked to better prognosis.

The concentrated nature of these critical features indicates the presence of a relatively compact biomarker signature in urine, thereby facilitating streamlined diagnostic applications. The PSI-MS dataset exhibits a more concentrated set of features, and thus the model RF-TabPFN-236 shows the best result of 98,75% classification accuracy. Though only a smaller number of features were removed, it still outperforms TabPFN on the unfilted feature set. So, combining TabPFN with feature selection is superior in our type of data. This pattern signifies a more complex and heterogeneous metabolic profile, likely attributable to the varied biological processes represented in serum. Consequently, the classification model must extract relevant signals from a wider range of metabolites to ensure the maintenance of high diagnostic accuracy.

The SHAP-based feature importance analysis provides interpretable insights into the model's decision-making process, facilitating the identification of biomarkers with high clinical relevance. These results highlight the flexibility of the Autoencoder-TabPFN model, which can adapt to the unique feature distributions of each dataset. This adaptability is crucial for enabling precise diagnostics across diverse metabolomic datasets. The improvement gained by using feature selection and compression is also evident in all classification metrics, as shown in Table 1.

Exploring the relationship between selected features and accuracy

We systematically varied the feature count using a random forest-based selection approach to evaluate the relationship between the number of selected features and classification accuracy. For the FI-TWIM-MS dataset, the results reveal that using only 80 features achieves near-optimal accuracy. Beyond 150 features, the accuracy plateaus and further increases in feature count introduce a mild risk of overfitting, particularly given the small sample size. This suggests that a smaller subset of highly informative features is sufficient to distinguish prostate and breast cancer cases from healthy controls. In contrast, the PSI-MS dataset exhibits a more pronounced dependency on feature count, with significant improvements in accuracy observed as the number of features increases. The hyperparameter optimization returns similar sizes of feature selection for all folds. In Figure 4 we analyse the distribution of importance among the 20 most important features in each dataset. For the FI-TWIM-MS dataset we find that the importance is more evenly distributed, while for the PS-MS dataset, the importance is more concentrated.

These findings highlight the importance of dataset-specific feature selection strategies. For the FI-TWIM-MS dataset, recombining existing features in the latent space yields the best results. For the higher-dimensional PSI-MS dataset, we found that pre-selecting the most valuable features, which have the most significant impact on performance, is more effective. This highlights the versatility of the proposed approach in optimising performance across diverse datasets.

Comparing the proposed model to other methods

To assess the effectiveness of the proposed Autoencoder/RF–TabPFN pipeline, we performed a benchmarking analysis against various deep learning and machine learning baseline models, using identical preprocessing and feature normalisation procedures. A nested 10-fold stratified cross-validation (CV) approach was employed to ensure fair comparison among the models and to support robust hyperparameter optimisation.

Performance on prostate datasets: In the analysis of the FI-TWIM-MS serum dataset, the proposed methodology achieved a Receiver Operating Characteristic Area Under the Curve (ROC-AUC) of 0.96 and an F1-score of 0.89 when employing tree-based feature pre-selection. This performance slightly surpasses that of the autoencoder-based compression variant, which recorded an ROC-AUC of 0.95 and an F1-score of 0.84. For the PSI-MS urine dataset, the same configuration attained an impressive ROC-AUC of 0.99, together with an F1-score

of 0.99. These results highlight the robustness of the method across different ionisation modalities.

Performance on the breast-cancer dataset: In the analysis of the MiniMaP breast-biopsy PSI-MS dataset, the autoencoder-based classifier achieved a Receiver Operating Characteristic Area Under the Curve (ROC-AUC) of 0.92, with an F1 score of 0.89. In comparison, the variant using random forest feature selection showed a marginal improvement, reaching an ROC-AUC of 0.93 and an F1 score of 0.90. These results support the model's ability to generalise effectively across different PSI-MS domains.

Baseline model comparison: Alternative deep learning baselines, including a compact one-dimensional convolutional neural network (1D-CNN), a multilayer perceptron (MLP), and an MLP-Surv architecture, were evaluated under consistent cross-validation conditions.

The 1D-CNN demonstrated impressive performance, achieving ROC-AUC scores of 0.94 (F1 score 0.87) on the FI-TWIM-MS dataset, 0.93 (F1 score 0.89) on the PSI-MS dataset, and 0.92 (F1 score 0.90) on the breast dataset. In comparison, the standard Multi-Layer Perceptron (MLP) attained ROC and F1 scores of 0.95 and 0.88 respectively on the FI-TWIM-MS dataset, and 0.95 and 0.82 on the PSI-MS dataset when using tree-based selection methods. Alternatively, using Autoencoder (AE)-based compression, the MLP achieved ROC and F1 scores of 0.919 and 0.90, and 0.925 and 0.86 for the respective datasets. On the breast dataset, AE-based compression resulted in ROC and F1 scores of 0.92 and 0.90. The MLP-Surv model showed competitive yet marginally lower performance, with ROC-AUC scores of 0.94 (F1 score 0.87) on the FI-TWIM-MS dataset, 0.93 and 0.86 on the PSI-MS dataset, and 0.92 and 0.88 on the breast dataset, using tree-based pre-selection methods. Notably, the AE-based preprocessing of features tended to outperform the tree-based pre-selection on the baseline methods.

The XGBoost classifier, using its built-in feature selection and evaluated through 10-fold cross-validation, achieved ROC-AUC and F1 score pairs of 0.91 / 0.83 for FI-TWIM-MS, 0.96 / 0.89 for PSI-MS, and 0.91 / 0.88 for breast cancer. Although XGBoost showed competitive performance, its metrics were consistently lower than those obtained with the proposed TabPFN-based pipeline.

Interpretation and scalability: TabPFN consistently demonstrated superior performance compared to traditional models across various datasets by effectively utilising prior-data fine-tuning and probabilistic feature reasoning, particularly in scenarios characterised by small sample sizes and high-dimensionality, which are typical in metabolomics. Unlike conventional

machine learning models such as Random Forests (RF) and XGBoost, which depend heavily on explicit feature engineering, TabPFN incorporates domain-agnostic priors and learns non-linear dependencies autonomously.

This advantage becomes increasingly evident as dataset complexity escalates, emphasising the scalability of deep foundation-model architectures for applications in metabolomics diagnostics. Figure 4 presents a summary of the comparative performance across all models and datasets, illustrating the superior distributions of ROC-AUC and F1-score achieved by the proposed Autoencoder/RF–TabPFN pipeline.

Classification by disease condition and experimental type

In the next step of the analysis, the performance of our model was verified using a consolidated dataset that combined the urine-based PSI-MS dataset and the serum-based FI-TWIM-MS dataset. To establish a unified feature space, we initially employed a Random Forest classifier to select the top 140 most significant features (ranked by their contribution to classification efficiency). The two datasets were then merged and normalized prior to the training and evaluation of the model.

As illustrated in Figure 5, the t-SNE projection of the resulting dataset reveals that samples predominantly cluster according to the analytical technique employed, rather than by clinical condition. This makes it more challenging to differentiate between healthy and diseased individuals than to distinguish between PSI-MS and FI-TWIM-MS sources.

Despite this challenge, the resulting RF–TabPFN-140 model demonstrated robust predictive performance, achieving an accuracy of 94%, a weighted F1-score of 0.94 and an ROC-AUC of 0.99.

Figure 5A presents the original two-dimensional t-SNE representation of the integrated feature space. Figure 5B highlights the sample correctly classified as belonging to the first or second dataset. We achieve a virtual 100% accuracy. Figure 5C illustrates classification outcomes based on both dataset origin and clinical condition. It is noteworthy that the model consistently classified samples accurately according to their dataset of origin, while classification based on health status proved more challenging, likely due to cross-platform variability. This highlights the need for improvements in the integration of different types of datasets to make use of a more unified approach. Due to the difference in the feature space additional batch correction methods would currently not be appropriate.

Examining the spectral data across datasets

To further evaluate the challenges of cancer diagnosis using metabolomic data, we analysed the group-averaged spectral profiles of diseased and healthy individuals for both datasets from both PSI-MS and FI-TWIM-MS. Figure 6 showcases these profiles, as well as both datasets. The raw mass spectra shared similar m/z ranges; however, subtle variations in intensity and peak distribution were observed.

The distinctions observed were not immediately apparent through conventional spectral inspection due to the complex nature and high dimensionality of the metabolomic profiles. It is essential to note that, although the mass ranges assessed were consistent, the spectral profiles generated by the two techniques displayed variations in resolution, baseline noise, and peak sharpness. These differences reflect fundamental discrepancies in ionisation mechanisms as well as in the sample matrices employed.

The model addresses this challenge by identifying non-linear relationships and utilizing subtle differences in the spectral data to enhance classification accuracy. For datasets, the spectral profiles of diseased and healthy individuals overlap even more extensively. The overlapping nature of these profiles makes it nearly impossible for manual or linear approaches to differentiate between the two groups effectively. Nonetheless, the proposed model achieves high accuracy, demonstrating its robustness in handling challenging datasets.

These findings highlight the importance of integrating deep learning with spectral analysis. The Autoencoder-TabPFN model excels at processing complex, noisy datasets, enabling reliable classification even when spectral profiles are visually indistinguishable. This capability is crucial for advancing metabolomics-based diagnostics, where the accurate interpretation of subtle spectral differences can significantly enhance disease detection and monitoring.

Conclusion

This study presents a novel diagnostic method that integrates an Autoencoder for dimensionality reduction, feature pre-selection via random forests and SHAP analysis, and a TabPFN classifier to analyse high-dimensional metabolomic data derived from PSI-MS (urine) and FI-TWIM-MS (serum) sources. The proposed approach demonstrated exceptional diagnostic efficacy in detecting prostate cancer, achieving classification accuracies of up to 98.75%, despite the inherent complexity and limited size of the datasets.

The integration of explainable artificial intelligence methodologies with deep learning has facilitated robust predictive capabilities alongside biological interpretability. Interestingly, significant prostate cancer-related biomarkers, including creatinine and TMAO, were consistently identified, thereby reinforcing the model's biological relevance. The autoencoder component adeptly minimised noise in high-dimensional input while retaining essential latent

features. Additionally, the pre-trained architecture of TabPFN ensured effective generalization, even in contexts of limited sample sizes.

This work highlights the potential of rapid, non-invasive diagnostic techniques employing ambient mass spectrometry methods, specifically PSI-MS and FI-TWIM-MS, in conjunction with advanced artificial intelligence models that can process heterogeneous and high-dimensional datasets. In contrast to conventional workflows that necessitate extensive sample preparation and lengthy chromatographic processes, the proposed approach demonstrates efficiency with minimal preprocessing, making it suitable for real-time applications in clinical settings.

Despite the promising results obtained, further validation through multi-institutional and longitudinal datasets is essential to establish reproducibility, particularly across various disease stages and diverse patient populations. Additionally, standardizing sample collection protocols, along with integrating clinical metadata, remains a critical step towards the routine implementation of these findings in clinical practice.

In summary, the Autoencoder/RF-TabPFN approach offers a scalable and interpretable approach for cancer diagnostics based on metabolomics. This framework establishes a foundation for future applications in precision medicine, not only within the field of oncology but also potentially across various metabolic disorders.

Availability

Data and code can be found in our github repository:
https://github.com/SvenHauns/metabolemic_classifier.git

Figures and Tables:

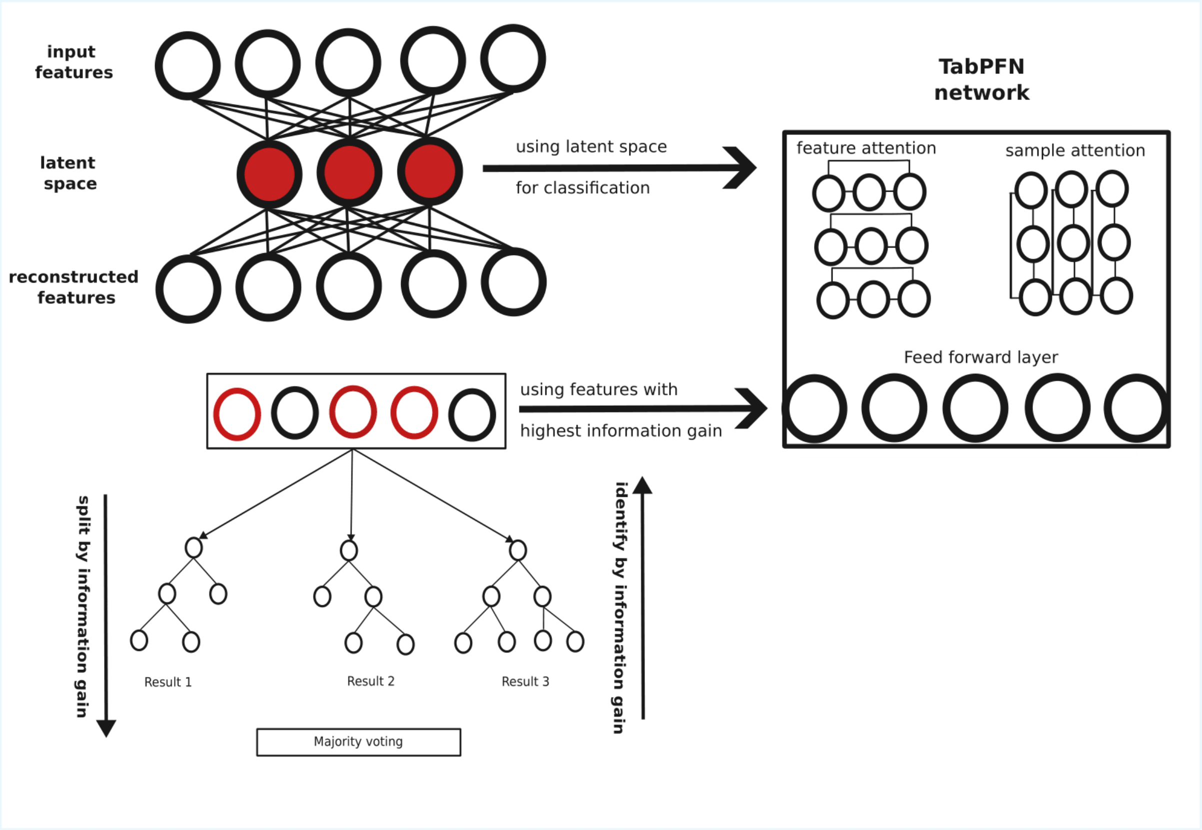

Figure 1. It shows the principle feature selection workflow employed to prevent overfitting. An autoencoder is used to create a latent dimension for classification. This helps with reducing redundant information and de-noises the data for further processing. Alternatively, we use the information gained based on random forests to assess which feature to select for further processing.

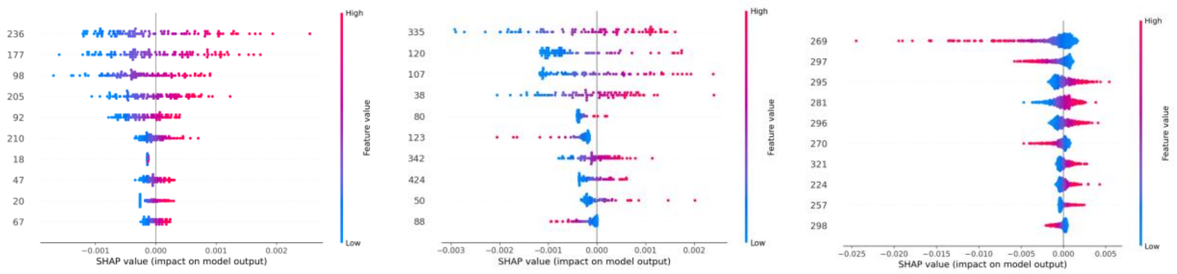

Figure 2 shows the most important input features determined by SHAP values on the two datasets. The most important features for both datasets are spread throughout the dataset, illustrating the importance of carefully selecting the input features to be used for classification later on. Low importance is shown in blue shades, high importance in red shades.

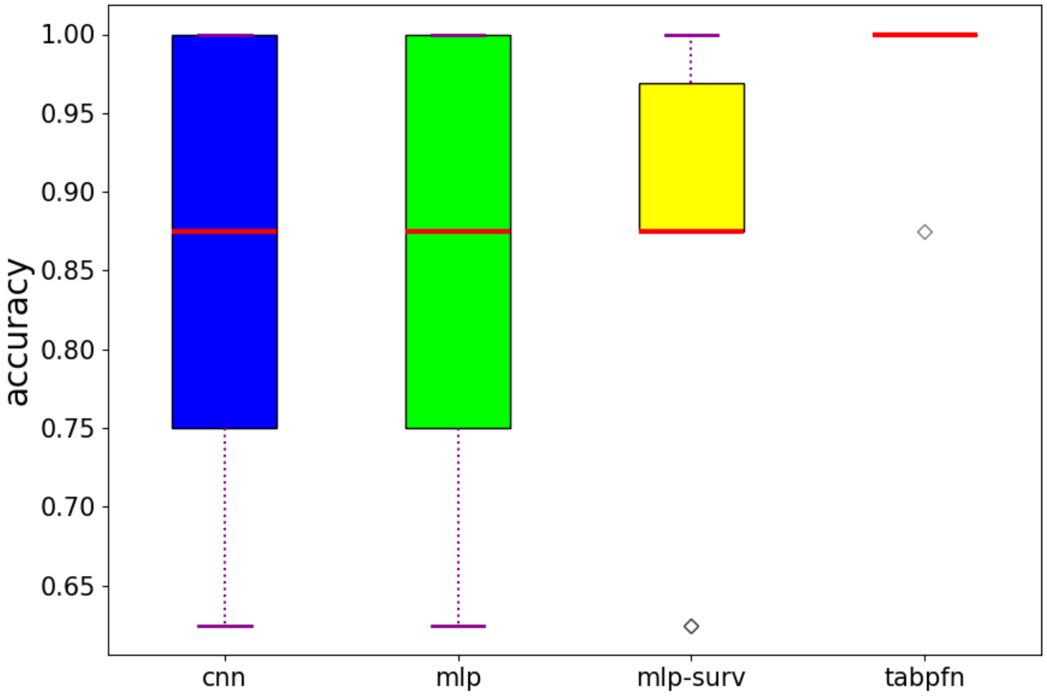

Figure 3. The figure compares the performance distribution of different baseline models on the PSI-MS dataset. For all models, we display the performance together with the best compression method used. Standard deep learning techniques perform best when combined with AE-based compression, while TabPFN achieves its highest performance with tree-based feature pre-selection. For all models, we perform hyperparameter optimization over the number of input features, starting with a minimum of 40 features and increasing in steps of 20 up to a maximum of 500 features. Shown are the performance of every outer CV.

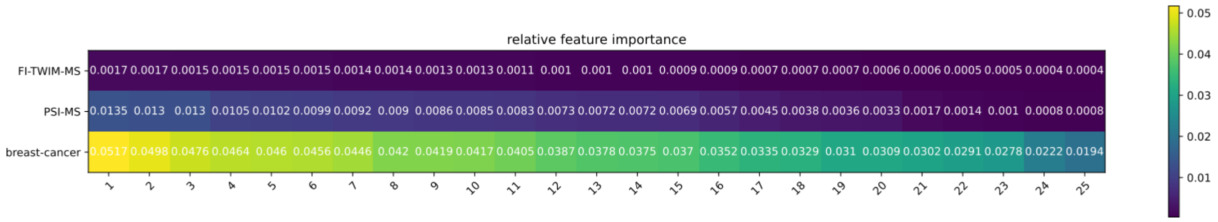

Figure 4: The heatmap illustrates the relative significance of the top 25 features from both datasets, as determined by scaled SHAP (Shapley Additive Explanations) values. Each cell represents the contribution of a feature to the model's prediction, with higher-intensity colors (yellow) denoting greater importance. In the FI-TWIM-MS dataset, the importance of features is more evenly distributed among the top 20 values. Conversely, the PSI-MS dataset exhibits a more concentrated distribution of feature importance across various variables, indicating a broader and more diffuse biomarker signature. The breast cancer dataset shows particularly

high values for the top 25 values, explaining its relative stability in performance regardless of feature selection. These observed patterns reflect technique-specific distinctions in metabolite detection, highlighting the adaptability of the proposed deep learning method in identifying relevant biomarkers across diverse experimental platforms.

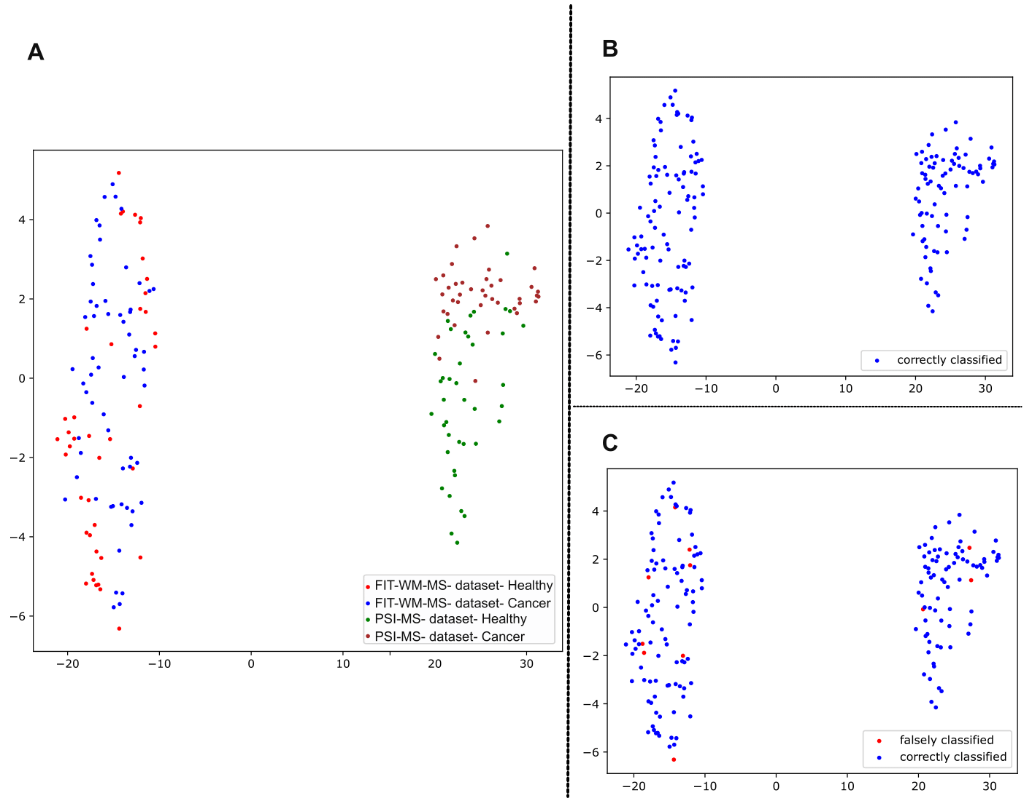

Figure 5. t-SNE plot of combined samples for both datasets used for the classification with AE-TabPFN on four classes (two datasets × two conditions). A) Shows the ground truth for 2 datasets and 2 conditions. B) shows the classification results only regarding classification in dataset 1 or dataset 2. We find that our approach can distinguish between both methods with 100% accuracy. C) shows the correctly and falsely classified samples of both datasets according to the ground truth shown in A).

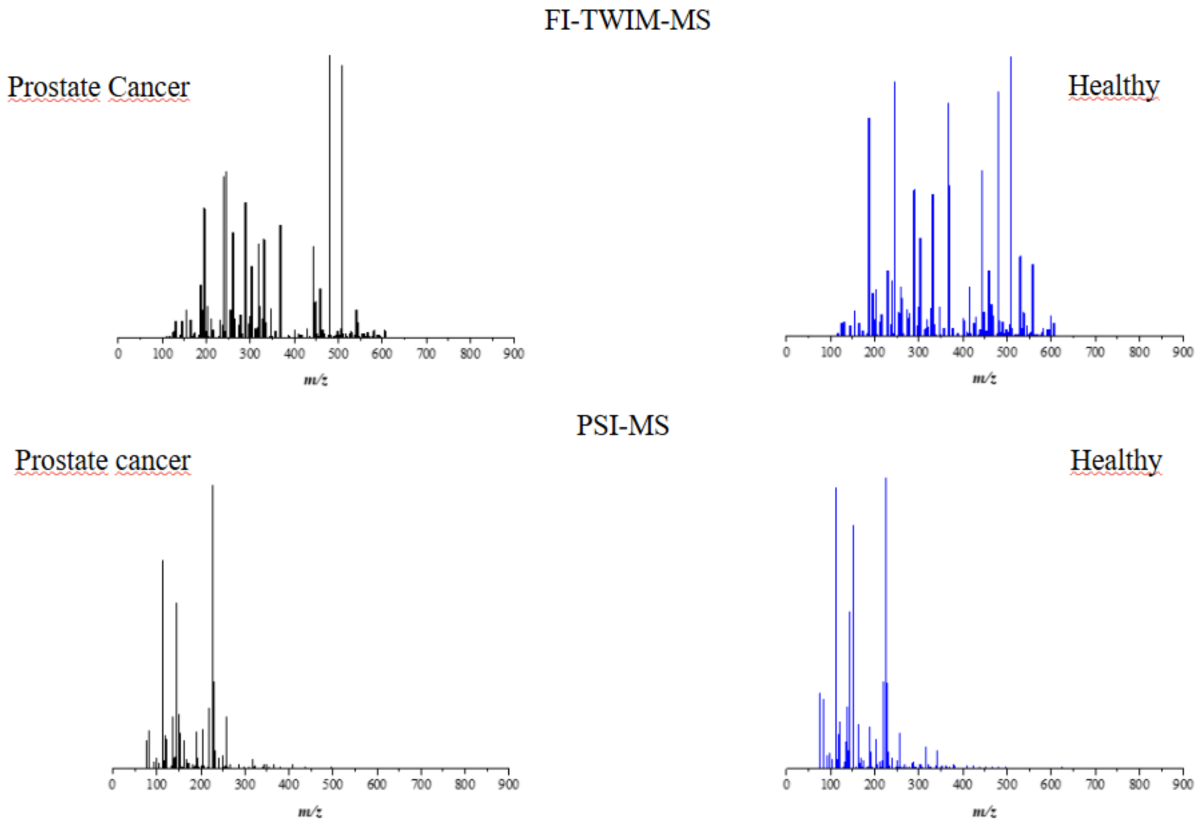

Figure 6. It presents the group-averaged metabolomic spectral profiles obtained from healthy controls and prostate cancer patients utilizing two advanced mass spectrometry techniques. Paper Spray Ionization Mass Spectrometry (PSI-MS) and Flow Injection–Two-Way Mirror Mass Spectrometry (FI-TWIM-MS). Although both techniques encompass the same mass-to-charge (m/z) range on the x-axis, their spectral profiles reveal distinct characteristics. PSI-MS and FI-TWIM-MS demonstrate notable differences in peak intensities and distributions. These variations reflect the differing influences of ionization efficiency, matrix effects, and the mechanisms of metabolite capture inherent to each method. Our method successfully identified subtle, non-linear patterns within the spectra, facilitating high-accuracy classification of patient groups, irrespective of variability associated with specific techniques.

experiment	TabPFN	RF/AE-TabPFN
FI-TWIM-MS-Dataset	Accuracy: 0.88<br>ROC-AUC: 0.93<br>F1: 0.88	Accuracy: 0.91<br>ROC-AUC: 0.95<br>F1:0.91
PSI-MS-Dataset	Accuracy: 0.975<br>ROC-AUC: 1.0<br>F1: 0.97	Accuracy:0.99<br>ROC-AUC: 1.0<br>F1:0.99
Breast cancer dataset	Accuracy:0.91<br>ROC-AUC 0.93<br>F1-score of 0.90	Accuracy:0.90<br>ROC-AUC 0.94<br>F1-score of 0.90
Combined datasets	Accuracy:NA[1]<br>ROC-AUC: NA<br>F1: NA	Accuracy:0.94<br>ROC-AUC: 0.99<br>F1: 0.94

Table 1: We see a general improvement in classification performance throughout all metrics when applying feature selection or transformation before using TabPFN. 1)The combination of both datasets needs preliminary feature selection, due to different feature dimensions. Therefore, TabPFN cannot be executed solely on the combined datasets.

Acknowledgments

The authors acknowledge support by the High Performance and Cloud Computing Group at the Zentrum für Datenverarbeitung of the University of Tübingen, the state of Baden-Württemberg through bwHPC and the German Research Foundation (DFG) through grant number INST 37/935-1 FUGG. This work was supported by the BMBF-funded de.NBI Cloud within the German Network for Bioinformatics Infrastructure (031A532B, 031A533A, 031A533B, 031A534A, 031A535A, 031A537A, 031A537B, 031A537C, 031A537D, 031A538A). The authors would also like to thank Mohammed Bin Rashid University of Medicine and Health Sciences (MBRU) and the reviewers for their valuable suggestions.

Funding

This study was supported by the Center for Applied and Translational Genomics (CATG), Mohammed Bin Rashid University of Medicine and Health Sciences (MBRU), Dubai Health, Dubai, United Arab Emirates. Internal grant MBRU, Dubai Health, College of Medicine grants MBRU-CM-RG2024-07 and MBRU-CM-RG2025-12. Deutsche Forschungsgemeinschaft grant BA 2168/23, Much More Than Defence: The Multiple Functions and Facets of CRISPR–Cas; Deutsche Forschungsgemeinschaft, grant BA 2168/25-1, Einluss von RNA-bindenden Proteinen und mRNA-Strukturen auf alternative Translation-Regulationsmechanismen im entzündlichen Tumorgeschehen. The article processing charge is funded by the Baden-Wuerttemberg Ministry of Science, Research and Art and the University of Freiburg in the funding programme Open Access Publishing.

Conflict of Interest

The authors declare no conflict of interest.

References

1. Bauer, M., et al. "Precision medicine and artificial intelligence: A pilot study on deep learning for cancer diagnostics." npj Precision Oncology, 5, 2021, doi: 10.1038/s41698-021-00173-8

2. Nicholson, J.K., & Lindon, J.C. "Systems biology: Metabonomics." Nature, 455, 1054–1056 (2008), doi:10.1038/4551054a

3. Silva MF, Carvalho TC, Rocha WFC, Vaz BG, Romão W. Molecularly imprinted polymer-coated paper as a substrate for highly sensitive analysis using paper spray mass spectrometry: quantification of metabolites in urine. *Analytical Methods,*2017; 9 (36), 5318–5324, doi: https://doi.org/10.1039/C7AY01648D.

4. Espy RD, Teunissen SF, Manicke NE, Ren Y, Ouyang Z, van Asten A, Cooks RG. Paper spray and extraction spray mass spectrometry for the direct and simultaneous quantification of eight drugs of abuse in whole blood. Anal Chem. 2014 Aug 5;86(15):7712-8. doi: 10.1021/ac5016408. Epub 2014 Jul 9. PMID: 24970379.

5. Sarkar D, Sinclair E, Lim SH, Walton-Doyle C, Jafri K, Milne J, Vissers JPC, Richardson K, Trivedi DK, Silverdale M, Barran P. Paper Spray Ionization Ion Mobility Mass Spectrometry of Sebum Classifies Biomarker Classes for the Diagnosis of Parkinson's Disease. JACS Au. 2022 Sep 7;2(9):2013-2022. doi: 10.1021/jacsau.2c00300. PMID: 36186554; PMCID: PMC9516698.

6. Mahmud I, Pinto FG, Rubio VY, Lee B, Pavlovich CP, Perera RJ, Garrett TJ. Rapid Diagnosis of Prostate Cancer Disease Progression Using Paper Spray Ionization Mass Spectrometry. Anal Chem. 2021 Jun 8;93(22):7774-7780. doi: 10.1021/acs.analchem.1c00943. Epub 2021 May 27. PMID: 34043339.

7. Pinto FG, Mahmud I, Rubio VY, Máquina ADV, Furtado Durans AF, Neto WB, Garrett TJ. Data-Driven Soft Independent Modeling of Class Analogy in Paper Spray Ionization Mass Spectrometry-Based Metabolomics for Rapid Detection of Prostate Cancer. Anal Chem. 2022 Feb 1;94(4):1925-1931. doi: 10.1021/acs.analchem.1c04004. Epub 2022 Jan 21. PMID: 35060703.

8. Huang YC, Chung HH, Dutkiewicz EP, Chen CL, Hsieh HY, Chen BR, Wang MY, Hsu CC. Predicting Breast Cancer by Paper Spray Ion Mobility Spectrometry Mass Spectrometry and Machine Learning. Anal Chem. 2020 Jan 21;92(2):1653-1657. doi: 10.1021/acs.analchem.9b03966. Epub 2019 Dec 10. PMID: 31809016.

9. Wang H, Manicke NE, Yang Q, Zheng L, Shi R, Cooks RG, Ouyang Z. Direct analysis of biological tissue by paper spray mass spectrometry. Anal Chem. 2011 Feb 15;83(4):1197-201. doi: 10.1021/ac103150a. Epub 2011 Jan 19. PMID: 21247069; PMCID: PMC3039116.

10.Zang X, Monge ME, Gaul DA, Fernández FM. Flow Injection-Traveling-Wave Ion Mobility-Mass Spectrometry for Prostate-Cancer Metabolomics. Anal Chem. 2018 Nov 20;90(22):13767-13774. doi: 10.1021/acs.analchem.8b04259. Epub 2018 Nov 8. PMID: 30379062.

11. Chetwynd AJ, David A. A review of nanoscale LC-ESI for metabolomics and its potential to enhance the metabolome coverage. Talanta. 2018 May 15;182:380-390. doi: 10.1016/j.talanta.2018.01.084. Epub 2018 Feb 5. PMID: 29501168.

12. Dunn WB, Broadhurst D, Begley P, Zelena E, Francis-McIntyre S, Anderson N, Brown M, Knowles JD, Halsall A, Haselden JN, Nicholls AW, Wilson ID, Kell DB, Goodacre R; Human Serum Metabolome (HUSERMET) Consortium. Procedures for large-scale metabolic profiling of serum and plasma using gas chromatography and liquid chromatography coupled to mass spectrometry. Nat Protoc. 2011 Jun 30;6(7):1060-83. doi: 10.1038/nprot.2011.335. PMID: 21720319.

13. Hollmann, Noah & Müller, Samuel & Purucker, Lennart & Krishnakumar, Arjun & Körfer, Max & Hoo, Shi & Schirrmeister, Robin & Hutter, Frank. (2025). Accurate predictions on small data with a tabular foundation model. Nature. 637. 319-326. 10.1038/s41586-024-08328-6.

14. Hong, Yuhui and Ye, Yuzhen and Tang, Haixu; Machine Learning in Small-Molecule Mass Spectrometry; Annual Review of Analytical Chemistry;1936-1335;2025;doi.org/10.1146/annurev-anchem-071224-08215

15. Caroline H. Johnson, Julijana Ivanisevic & Gary Siuzdak; Metabolomics: beyond biomarkers and towards mechanisms; Nature Reviews Molecular Cell Biology; 17, 451–459 (2016); doi:10.1038/nrm.2016.25

16. Huang, HC., Chung, HH., Yu, JY. *et al.* Development and multicenter validation of on-site breast cancer diagnosis using paper spray ionization miniature mass spectrometry. *Commun Med* **5**, 259 (2025). https://doi.org/10.1038/s43856-025-00930-7

17. Katzman, J.L., Shaham, U., Cloninger, A. *et al.* DeepSurv: personalized treatment recommender system using a Cox proportional hazards deep neural network. *BMC Med Res Methodol* **18**, 24 (2018). https://doi.org/10.1186/s12874-018-0482-1

18. Renata de Freitas Saito, Luciana Nogueira de Sousa Andrade, Silvina Odete Bustos, Roger Chammas; Phosphatidylcholine-Derived Lipid Mediators: The Crosstalk Between Cancer Cells and Immune Cells;eCollection 2022; 10.3389/fimmu.2022.768606

19. Menglin Cheng, Zaver M Bhujwalla, Kristine Glunde; Targeting Phospholipid Metabolism in Cancer; Front Oncol; 2016:6:266. doi: 10.3389/fonc.2016.00266

20. Egidio Iorio, Delia Mezzanzanica, Paola Alberti, Francesca Spadaro, Carlo Ramoni, Sandra D'Ascenzo, Danilo Millimaggi, Antonio Pavan, Vincenza Dolo, Silvana Canevari, Franca Podo; Alterations of choline phospholipid metabolism in ovarian tumor progression; Cancer Res; 2005:9369-76. Doi: 10.1158/0008-5472.CAN-05-1146

21. Renata de Freitas Saito, Luciana Nogueira de Sousa Andrade, Silvina Odete Bustos, Roger Chammas; Phosphatidylcholine-Derived Lipid Mediators: The Crosstalk Between Cancer Cells and Immune Cells; Front Immunol; 2022:13:768606. doi: 10.3389/fimmu.2022.768606.

22. Menglin Cheng, Zaver M Bhujwalla, Kristine Glunde; Targeting Phospholipid Metabolism in Cancer; Front Oncol; 2016:6:266. doi: 10.3389/fonc.2016.00266.

23. Egidio Iorio, Delia Mezzanzanica, Paola Alberti, Francesca Spadaro, Carlo Ramoni, Sandra D'Ascenzo, Danilo Millimaggi, Antonio Pavan, Vincenza Dolo, Silvana Canevari, Franca Podo; Alterations of choline phospholipid metabolism in ovarian tumor progression; Cancer Res; 2005;65(20):9369-76. doi: 10.1158/0008-5472.CAN-05-1146

24. Lara P Fernández, Marta Gómez de Cedrón, Ana Ramírez de Molina; Alterations of Lipid Metabolism in Cancer: Implications in Prognosis and Treatment; Front Oncol; 2020 Oct 28:10:577420. doi: 10.3389/fonc.2020.577420

25. Chunyu Li, Fei Wang, Lili Cui, Shaoxin Li, Junyu Zhao, Lin Liao; Association between abnormal lipid metabolism and tumor; Front Endocrinol (Lausanne); 2023:14:1134154. doi: 10.3389/fendo.2023.1134154

26. Renshuai Zhang, Jingsen Meng, Shanbo Yang, Wenjing Liu, Lingyu Shi, Jun Zeng, Jing Chang, Bing Liang, Ning Liu, Dongming Xing; Recent Advances on the Role of ATGL in Cancer; Front Oncol; 2022:12:944025. doi: 10.3389/fonc.2022.944025

Figure Click here to access/download;Figure;Figure_and_table.pdf

Figures and Tables:

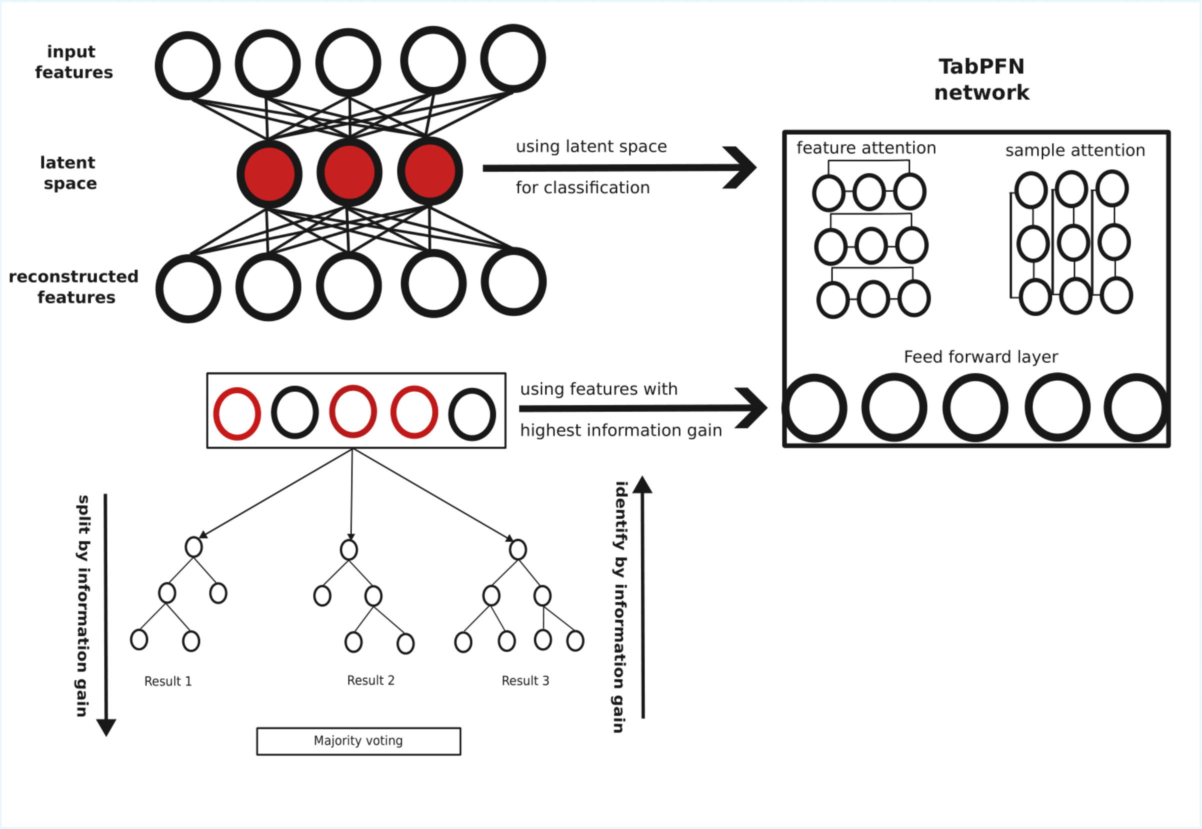

Figure 1. It shows the principle feature selection workflow employed to prevent overfitting. An autoencoder is used to create a latent dimension for classification. This helps with reducing redundant information and de-noises the data for further processing. Alternatively, we use the information gained based on random forests to assess which feature to select for further processing.

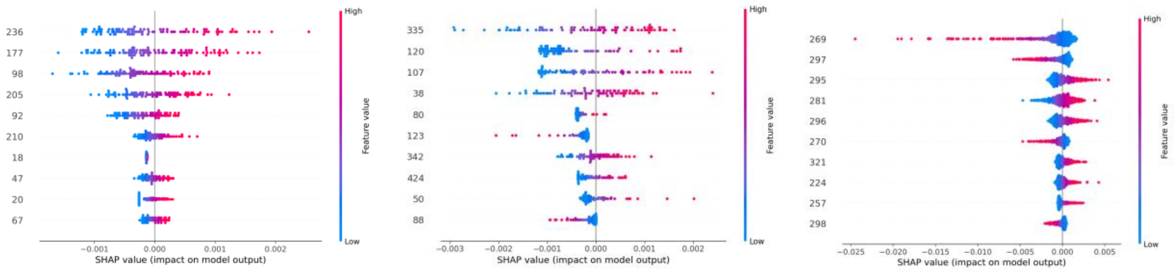

Figure 2 shows the most important input features determined by SHAP values on the two datasets. The most important features for both datasets are spread throughout the dataset, illustrating the importance of carefully selecting the input features to be used for classification later on. Low importance is shown in blue shades, high importance in red shades.

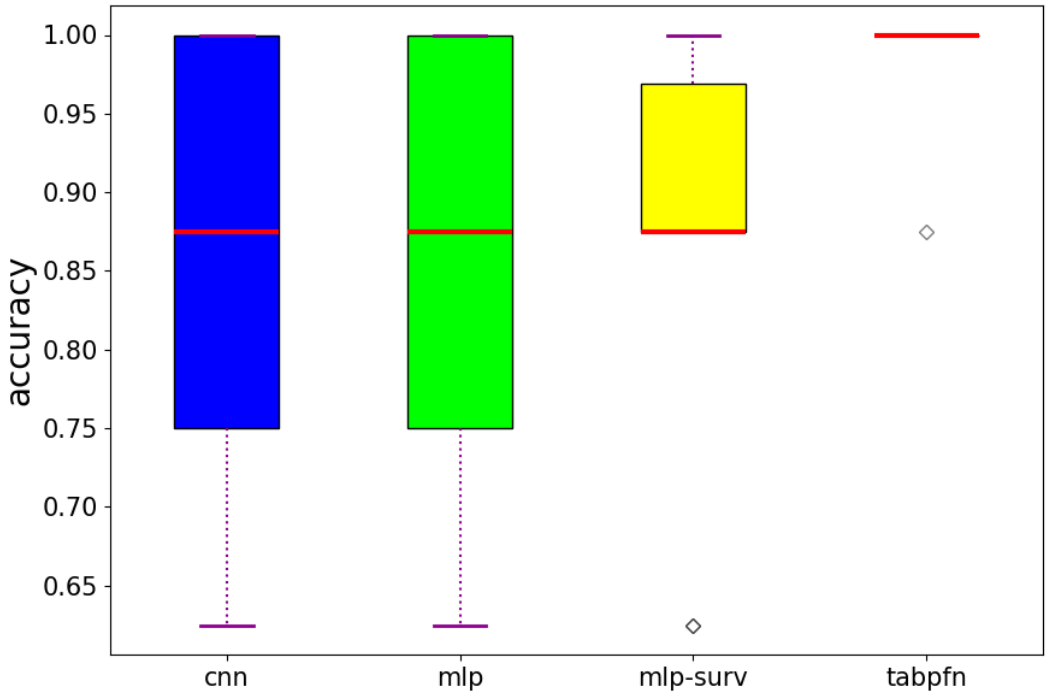

Figure 3. The figure compares the performance distribution of different baseline models on the PSI-MS dataset. For all models, we display the performance together with the best compression method used. Standard deep learning techniques perform best when combined with AE-based compression, while TabPFN achieves its highest performance with tree-based feature pre-selection. For all models, we perform hyperparameter optimization over the number of input features, starting with a minimum of 40 features and increasing in steps of 20 up to a maximum of 500 features. Shown are the performance of every outer CV.

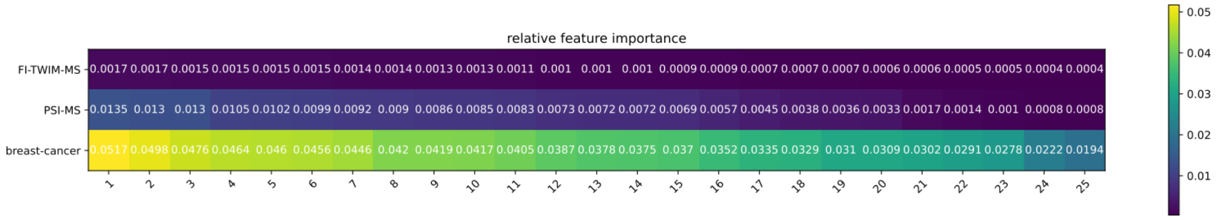

Figure 4: The heatmap illustrates the relative significance of the top 25 features from both datasets, as determined by scaled SHAP (Shapley Additive Explanations) values. Each cell represents the contribution of a feature to the model's prediction, with higher-intensity colors (yellow) denoting greater importance. In the FI-TWIM-MS dataset, the importance of features is more evenly distributed among the top 20 values. Conversely, the PSI-MS dataset exhibits a more concentrated distribution of feature importance across various variables, indicating a broader and more diffuse biomarker signature. The breast cancer dataset shows particularly high values for the top 25 values, explaining its relative stability in performance regardless of feature selection. These observed patterns reflect technique-specific distinctions in metabolite detection, highlighting the adaptability of the proposed deep learning method in identifying relevant biomarkers across diverse experimental platforms.

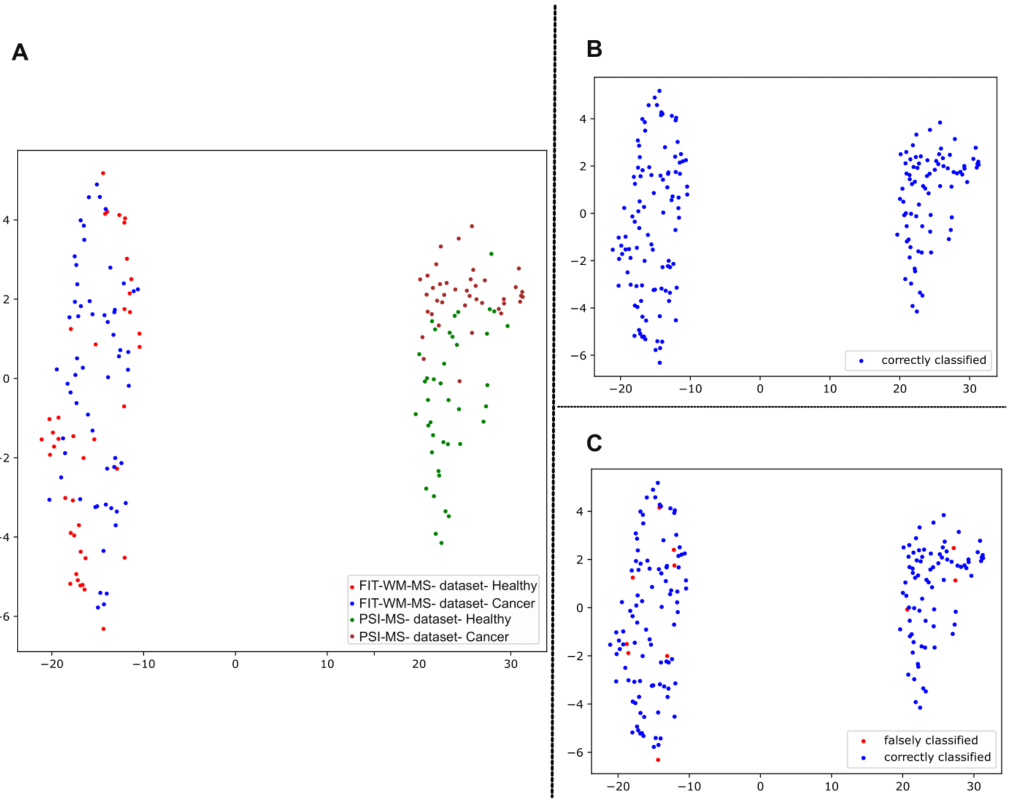

Figure 5. t-SNE plot of combined samples for both datasets used for the classification with AE-TabPFN on four classes (two datasets × two conditions). A) Shows the ground truth for 2 datasets and 2 conditions. B) shows the classification results only regarding classification in dataset 1 or dataset 2. We find that our approach can distinguish between both methods with

100% accuracy. C) shows the correctly and falsely classified samples of both datasets according to the ground truth shown in A).

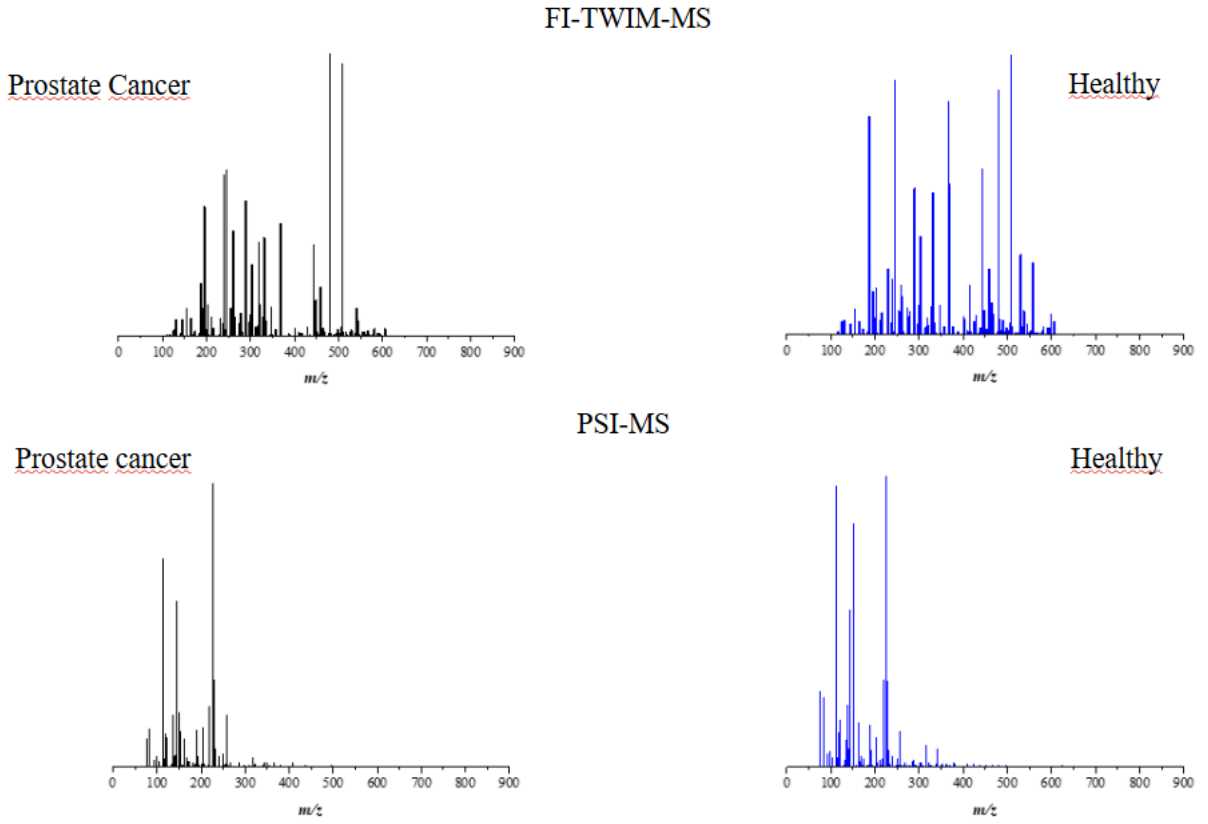

Figure 6. It presents the group-averaged metabolomic spectral profiles obtained from healthy controls and prostate cancer patients utilizing two advanced mass spectrometry techniques. Paper Spray Ionization Mass Spectrometry (PSI-MS) and Flow Injection–Two-Way Mirror Mass Spectrometry (FI-TWIM-MS). Although both techniques encompass the same mass-to-charge (m/z) range on the x-axis, their spectral profiles reveal distinct characteristics. PSI-MS and FI-TWIM-MS demonstrate notable differences in peak intensities and distributions. These variations reflect the differing influences of ionization efficiency, matrix effects, and the mechanisms of metabolite capture inherent to each method. Our method successfully identified subtle, non-linear patterns within the spectra, facilitating high-accuracy classification of patient groups, irrespective of variability associated with specific techniques.

experiment	TabPFN	RF/AE-TabPFN
FI-TWIM-MS-Dataset	Accuracy: 0.88<br>ROC-AUC: 0.93<br>F1: 0.88	Accuracy: 0.91<br>ROC-AUC: 0.95<br>F1:0.91
PSI-MS-Dataset	Accuracy: 0.975<br>ROC-AUC: 1.0<br>F1: 0.97	Accuracy:0.99<br>ROC-AUC: 1.0<br>F1:0.99
Breast cancer dataset	Accuracy:0.91<br>ROC-AUC 0.93<br>F1-score of 0.90	Accuracy:0.90<br>ROC-AUC 0.94<br>F1-score of 0.90
Combined datasets	Accuracy:NA[1]<br>ROC-AUC: NA<br>F1: NA	Accuracy:0.94<br>ROC-AUC: 0.99<br>F1: 0.94

Table 1: We see a general improvement in classification performance throughout all metrics when applying feature selection or transformation before using TabPFN. 1)The combination of both datasets needs preliminary feature selection, due to different feature dimensions. Therefore, TabPFN cannot be executed solely on the combined datasets.

GigaScience

Dear Editorial Borad

I’m pleased to submit our manuscript, titled ““Autoencoder/RF–TabPFN for Cross-Cancer Metabolomics: Prostate and Breast Cancer Diagnosis Using PSI-MS and FI-TWIM-MS,” for consideration as an article in the GigaScience. This manuscript has not been published previously and is not under consideration for publication elsewhere.

This manuscript presents an AI-driven framework combining an Autoencoder for unsupervised feature compression, Random Forest and SHAP for feature selection, and TabPFN—a foundation model based on pretrained transformers—for classification. We applied this method to two metabolomic datasets derived from noninvasive platforms: PSI-MS, FI-TWIM-MS and, focusing on the diagnosis of breast and prostate cancers.

Our results demonstrate that the proposed model achieves up to 98% classification accuracy, significantly outperforming traditional models such as Random Forest and SVM across metrics including F1-score, recall, and precision. The model also successfully identified relevant metabolites such as creatinine and TMAO, showing biological consistency with known prostate cancer markers. Additionally, SHAP-based feature analysis and t-SNE visualizations provide interpretability and confirm the distinct spectral patterns between patient groups.

This work is well-aligned with the aims of the GigaScience, as it:

- Applies **foundation models (TabPFN)** to real-world biomedical datasets
- Demonstrates the power of **deep learning in small-sample, high-dimensional settings**
- Provides a **clinically relevant, noninvasive diagnostic application** using AI-enhanced metabolomics
- Bridges **multiple data sources** and technologies to support generalizable precision diagnostics

We believe this study offers valuable insights into the integration of AI and foundation models in biomedicine and would be of significant interest to your readership.

Thank you for considering our manuscript.

Sincerely yours,

Dr. Omer Alkhnbashi

On behalf of all co-authors
