# Supplementary material for: Autoencoder/RandomForest–TabPFN for cross-cancer metabolomics: prostate and breast cancer diagnosis using paper spray and ion mobility-mass spectrometry techniques
Source: Gigascience. 2026 May 7;15:giag053. doi: 10.1093/gigascience/giag053 (PMC13251742; doi:10.1093/gigascience/giag053)
Supplement: giag053_Authors_Response_To_Reviewer_Comments_original_submission [file giag053_authors_response_to_reviewer_comments_original_submission.pdf]

Author's Response To Reviewer Comments

======================== Reviewer #1: ==

This study presents an innovative predictive method that integrates an autoencoder, random forest-based feature selection, and a tabular prior-data fitted network (TabPFN) to achieve high diagnostic accuracy from the metabolomic data.

- There are two types of cancers in this article, as the title claims, but only prostate cancer was introduced at the beginning of the Abstract and the Introduction. How about breast cancer?
Author's response: We thank the reviewer for his comment. We clarified it accordingly

- It is recommended to shorten the related content of FI-TWIM-MS and PSI-MS in the introduction. From my perspective, the key point lies in the predictive method
Author's response: We thank the reviewer for his comment. We shortened the introduction accordingly.

- What does the red line in Figure 3 refer to? It is a little awkward from a statistical view
Author's response: We thank the reviewer for his comment. The red line refers to the median value. We changed the figure accordingly.

- It is better to identify the metabolites according to the selected features
Author's response: We thank the reviewer for his comment. We thank the reviewer for this insightful and entirely valid comment. We agree that identifying metabolites are essential for establishing a comprehensive biological interpretation. However, the primary aim of the present study was to demonstrate a proof of concept that advanced artificial intelligence architectures—specifically, the Autoencoder/RF–TabPFN pipeline—can effectively model and classify heterogeneous metabolomics datasets from different analytical platforms (PSI-MS, FI-TWIM-MS, and MiniMaP PSI-MS) and various biological matrices (urine, serum, and tissue). Therefore, our focus was methodological: to evaluate whether a single, unified AI framework can generalise across these diverse data sources.

- More related advances on metabolomics and machine learning (https://doi.org/10.1002/VIW.20240031; Nature Sustainability 2024, 7, 602) should be included and discussed.
Author's response: We thank the reviewer for his comment. We added the referred article

- Please clarify the novelty of this work in the Conclusion section (or other place) by comparing with the art-of-state techniques in metabolic data analysis.
Author's response: We thank the reviewer for his comment. We clarified the novelty accordingly in the conclusion

======================== Reviewer #2: ==

Thank you for the opportunity to review this manuscript Autoencoder/RF-TabPFN for Cross-Cancer Metabolomics: Prostate and Breast Cancer Diagnosis Using PSI-MS and FI-TWIM-MS (GIGA-D-25-00458). Overall, the study addresses a timely and relevant methodological problem in metabolomics-driven diagnostics: how to achieve accurate classification from high-dimensional, small-sample datasets while maintaining interpretability and scalability. Overall, the study addresses a timely and relevant methodological problem in metabolomics-driven diagnostics: how to achieve accurate classification from

high-dimensional, small-sample datasets while maintaining interpretability and scalability.
While the study is promising, several major issues need to be addressed to strengthen the conclusions and overall impact of the work

- Risk of over-interpretation given limited sample sizes.
Although nested cross-validation is appropriately applied, the prostate cancer datasets remain relatively small (n≈80-100). The very high performance metrics reported (e.g., ROC-AUC ≈1.0) raise concerns about potential optimistic bias. The authors should more explicitly discuss the statistical uncertainty and variance across folds, and temper claims regarding "exceptional" or "near-perfect" diagnostic accuracy.
Author’s response: We thank the reviewer for his comment. We agree with the importance of being careful when interpreting performance on a small dataset. To improve interpretability we added the variance to the evaluation

- Clarification of cross-cancer generalizability
The breast cancer dataset differs substantially from the prostate datasets in tissue type, sample size, feature composition, and class balance. While the results are encouraging, it is not entirely clear whether the observed performance reflects true biological generalizability or simply the strong signal present in the breast biopsy dataset. This distinction should be clarified in the Discussion.
Author’s response: We thank the reviewer for his comment. We clarified the distinction in the dataset

- Biological interpretation remains limited
Although SHAP analysis is performed, most features are discussed at the level of m/z ranges rather than confidently annotated metabolites. While full metabolite identification may be beyond the scope of a Technical Note, the authors should clarify this limitation and avoid implying mechanistic insights that are not directly supported by the data.
Author’s response: We thank the reviewer for his comment. We fully agree that the practical usability and access to computational frameworks are vital for translating advances in bioinformatics into real clinical and research benefits. Although the current study mainly focuses on developing and validating a proof-of-concept AI framework (Autoencoder/RF–TabPFN) for cross-platform metabolomics analysis, we recognise the importance of ensuring that this approach remains accessible to non-technical users, including clinicians and researchers in metabolomics and oncology. Therefore, we released all code, trained model weights, and example data in an open-access GitHub repository to ensure complete transparency and reproducibility. We are also developing a web-based dashboard and a lightweight standalone tool (in Python/Streamlit) that will allow users to upload metabolomics datasets, apply preprocessing, and obtain classification and feature-importance outputs via the trained Autoencoder/RF–TabPFN pipeline.

- Clinical translation claims should be moderated
Statements suggesting near-term clinical or point-of-care deployment are premature without external prospective validation, standardized preprocessing, and integration with clinical metadata. These limitations should be more explicitly acknowledged in the Conclusion.
Author’s response: We thank the reviewer for his comment. We agree that claims about clinical application should only be made carefully. We now augmented the conclusion by stating so.

- Title readability and use of abbreviations
The title contains multiple technical abbreviations (e.g., AE, RF, TabPFN, PSI-MS, FI-TWIM-MS), which may hinder readability for a broad interdisciplinary audience. I recommend reducing the number of abbreviations in the title, or spelling out at least the less widely known methods, to improve accessibility and clarity.
Author’s response: We thank the reviewer for his comment. We adapted the title accordingly

- Some terminology is used inconsistently (e.g., "FI-TWIM-MS" vs. "FI-TWIM"). Standardizing nomenclature throughout would improve clarity.
Author’s response: We thank the reviewer for his comment. The terminology should be more consistent now.

- The description of TabPFN's ability to model "temporal dependencies" is potentially misleading, as the datasets are not longitudinal. This phrasing should be revised
Author's response: We thank the reviewer for his comment. We revised the phrasing accordingly.

- Figures are generally clear, but some captions (e.g., Figures 3-5) could be shortened and made more focused on the key message.
Author's response: We thank the reviewer for his comment. We adjusted the captions accordingly.

- Typographical and grammatical issues are minor but present and should be corrected during revision
Author's response: We thank the reviewer for his comment. We improved the paper accordingly

- Figure 6 -> there are still underlined headers
Author's response: We thank the reviewer for his comment. We removed it.

Cookie Preference Center

We use cookies which are necessary to make our site work. We may also use additional cookies to analyze, improve and personalize our content and your digital experience. For more information, see our Cookie Policy.

You may choose not to allow some types of cookies. However, blocking some types may impact your experience of our site and the services we are able to offer. See the different category headings below to find out more or change your settings.

Allow all

Manage Consent Preferences

Strictly Necessary Cookies

Always active

These cookies are necessary for the website to function and cannot be switched off in our systems. They are usually only set in response to actions made by you which amount to a request for services, such as setting your privacy preferences, logging in or

filling in forms. You can set your browser to block or alert you about these cookies, but some parts of the site will not then work.

Functional Cookies

These cookies enable the website to provide enhanced functionality and personalisation. They may be set by us or by third party providers whose services we have added to our pages. If you do not allow these cookies then some or all of these services may not function properly.

Performance Cookies

These cookies allow us to count visits and traffic sources so we can measure and improve the performance of our site. They help us to know which pages are the most and least popular and see how visitors move around the site.

Targeting Cookies

These cookies may be set through our site by our advertising partners. They may be used by those companies to build a profile of your interests and show you relevant adverts on other sites. If you do not allow these cookies, you will experience less targeted advertising.

Cookie List

Clear

Apply Cancel

Consent Leg.Interest

Confirm my choices

Power
