# Supplementary material for: Autoencoder/RandomForest–TabPFN for cross-cancer metabolomics: prostate and breast cancer diagnosis using paper spray and ion mobility-mass spectrometry techniques
Source: Gigascience. 2026 May 7;15:giag053. doi: 10.1093/gigascience/giag053 (PMC13251742; doi:10.1093/gigascience/giag053)
Supplement: giag053_Reviewer_2_Report_original_submission — Reviewer 2 -- 1/11/2026 [file giag053_reviewer_2_report_original_submission.pdf]

Reviewer Report

Title: Autoencoder/RandomForestâ€"TabPFN for Cross-Cancer Metabolomics: Prostate and Breast Cancer Diagnosis Using Paper Spray and Ion Mobility-Mass Spectrometry Techniques

Version: Original Submission **Date:** 1/11/2026

Reviewer name: Reviewer 2

Reviewer Comments to Author:

Thank you for the opportunity to review this manuscript Autoencoder/RF-TabPFN for Cross-Cancer Metabolomics: Prostate and Breast Cancer Diagnosis Using PSI-MS and FI-TWIM-MS (GIGA-D-25-00458). Overall, the study addresses a timely and relevant methodological problem in metabolomics-driven diagnostics: how to achieve accurate classification from high-dimensional, small-sample datasets while maintaining interpretability and scalability. Overall, the study addresses a timely and relevant methodological problem in metabolomics-driven diagnostics: how to achieve accurate classification from high-dimensional, small-sample datasets while maintaining interpretability and scalability.
While the study is promising, several major issues need to be addressed to strengthen the conclusions and overall impact of the work.
Major Comments
1. Risk of over-interpretation given limited sample sizes
Although nested cross-validation is appropriately applied, the prostate cancer datasets remain relatively small (nâ‰ˆ80-100). The very high performance metrics reported (e.g., ROC-AUC â‰ˆ1.0) raise concerns about potential optimistic bias. The authors should more explicitly discuss the statistical uncertainty and variance across folds, and temper claims regarding "exceptional" or "near-perfect" diagnostic accuracy.
2. Clarification of cross-cancer generalizability
The breast cancer dataset differs substantially from the prostate datasets in tissue type, sample size, feature composition, and class balance. While the results are encouraging, it is not entirely clear whether the observed performance reflects true biological generalizability or simply the strong signal present in the breast biopsy dataset. This distinction should be clarified in the Discussion.
3. Biological interpretation remains limited
Although SHAP analysis is performed, most features are discussed at the level of m/z ranges rather than confidently annotated metabolites. While full metabolite identification may be beyond the scope of a Technical Note, the authors should clarify this limitation and avoid implying mechanistic insights that are not directly supported by the data.
4. Clinical translation claims should be moderated
Statements suggesting near-term clinical or point-of-care deployment are premature without external prospective validation, standardized preprocessing, and integration with clinical metadata. These limitations should be more explicitly acknowledged in the Conclusion.
5. Title readability and use of abbreviations
The title contains multiple technical abbreviations (e.g., AE, RF, TabPFN, PSI-MS, FI-TWIM-MS), which

may hinder readability for a broad interdisciplinary audience. I recommend reducing the number of abbreviations in the title, or spelling out at least the less widely known methods, to improve accessibility and clarity.
Minor Comments
1. Some terminology is used inconsistently (e.g., "FI-TWIM-MS" vs. "FI-TWIM"). Standardizing nomenclature throughout would improve clarity.
2. The description of TabPFN's ability to model "temporal dependencies" is potentially misleading, as the datasets are not longitudinal. This phrasing should be revised.
3. Figures are generally clear, but some captions (e.g., Figures 3-5) could be shortened and made more focused on the key message.
4. Typographical and grammatical issues are minor but present and should be corrected during revision.

Methods

Are the methods appropriate to the aims of the study, are they well described, and are necessary controls included? Choose an item.

Conclusions

Are the conclusions adequately supported by the data shown? Choose an item.

Reporting Standards

Does the manuscript adhere to the journal's guidelines on minimum standards of reporting? Choose an item.

Choose an item.

Statistics

Are you able to assess all statistics in the manuscript, including the appropriateness of statistical tests used? Choose an item.

Quality of Written English

Please indicate the quality of language in the manuscript: Choose an item.

Declaration of Competing Interests

Please complete a declaration of competing interests, considering the following questions:

- Have you in the past five years received reimbursements, fees, funding, or salary from an organisation that may in any way gain or lose financially from the publication of this manuscript, either now or in the future?

- Do you hold any stocks or shares in an organisation that may in any way gain or lose financially from the publication of this manuscript, either now or in the future?
- Do you hold or are you currently applying for any patents relating to the content of the manuscript?
- Have you received reimbursements, fees, funding, or salary from an organization that holds or has applied for patents relating to the content of the manuscript?
- Do you have any other financial competing interests?
- Do you have any non-financial competing interests in relation to this paper?

If you can answer no to all of the above, write 'I declare that I have no competing interests' below. If your reply is yes to any, please give details below.

I declare that I have no competing interests

I agree to the open peer review policy of the journal. I understand that my name will be included on my report to the authors and, if the manuscript is accepted for publication, my named report including any attachments I upload will be posted on the website along with the authors' responses. I agree for my report to be made available under an Open Access Creative Commons CC-BY license (http://creativecommons.org/licenses/by/4.0/). I understand that any comments which I do not wish to be included in my named report can be included as confidential comments to the editors, which will not be published.

Choose an item.

To further support our reviewers, we have joined with Publons, where you can gain additional credit to further highlight your hard work (see: https://publons.com/journal/530/gigascience). On publication of this paper, your review will be automatically added to Publons, you can then choose whether or not to claim your Publons credit. I understand this statement.

Yes Choose an item.
